# Uncovering cantharidin’s mechanism for cholangiocarcinoma treatment using patient-derived tumor organoids

**DOI:** 10.1016/j.isci.2025.114136

**Published:** 2025-11-19

**Authors:** Pinsheng Han, Libo Wang, Liuyang Zhu, Wen Tong, Sen Liu, Tianze Wang, Tianrun Yang, ZhenZhen Li, Xiaolei Zhou, Tianyu Zhao, Tao Cui, Long Yang, Ze Wang, Yamin Zhang

**Affiliations:** 1 School of Medicine, Nankai University, Tianjin 300071, China; 2 State Key Laboratory of Druggability Evaluation and Systematic Translational Medicine, Tianjin Institute of Pharmaceutical Research, Tianjin 300000, China; 3 Department of Hepatobiliary Surgery, Tianjin First Central Hospital, School of Medicine, Nankai University, Tianjin 300384, China; 4 First Central Hospital of Tianjin Medical University, Tianjin 300192, China; 5 Department of Pharmacology, Shenyang Pharmaceutical University, Shenyang 110016, China; 6 Hefei Tianhui Biotechnology Co., Ltd., Hefei 230000, China

**Keywords:** Therapy, Clinical pharmacy, Cancer

## Abstract

This study investigates the antitumor efficacy of cantharidin against cholangiocarcinoma (CCA) using patient-derived organoids (PDOs) that faithfully replicate the histological and genomic features of original tumors. Results demonstrate that cantharidin effectively inhibits CCA PDO growth, showing comparable or superior efficacy to conventional chemotherapeutics such as cisplatin, though slightly less potent than gemcitabine or Adriamycin. Critically, drug sensitivity in PDOs correlated perfectly with clinical responses in five patients, validating the model’s predictive relevance. Mechanistic studies revealed that cantharidin suppresses proliferation and induces apoptosis primarily through downregulation of the p-ERK1/2-c-Fos signaling pathway, both *in vitro* and in patient-derived organoids-based xenografts. These effects were reversible upon treatment with a p-ERK agonist, confirming pathway specificity. The study highlights cantharidin’s potential as a targeted therapeutic agent in CCA and underscores the utility of PDOs in personalized drug screening and mechanistic investigation.

## Highlights

• Cantharidin shows potent antitumor efficacy in cholangiocarcinoma PDOs
• PDOs' drug sensitivity perfectly correlates with clinical responses in patients
• Cantharidin suppresses proliferation and induces apoptosis in cholangiocarcinoma
• The antitumor mechanism involves downregulation of the p-ERK1/2-c-Fos pathway

## Graphical abstract

**Figure:**
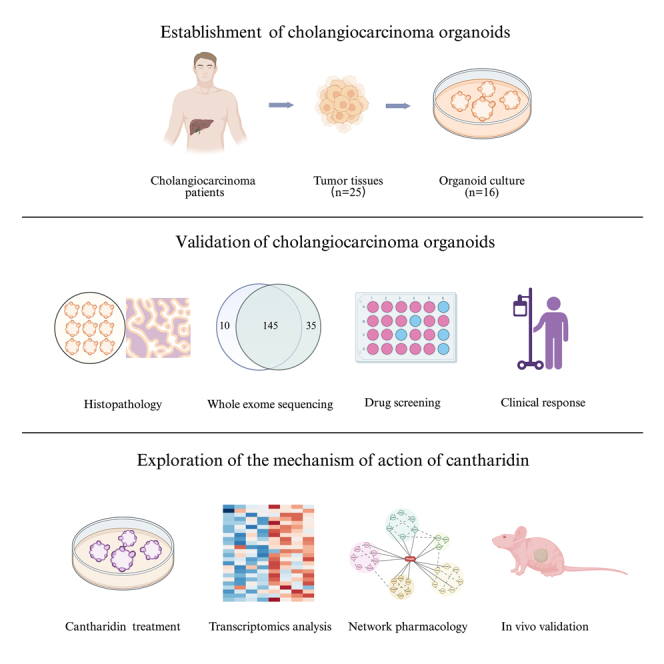

## Introduction

Cholangiocarcinoma (CCA) is a highly malignant tumor originating from the epithelial cells of the biliary ducts. It is classified into three major types based on the location of the cancer: intrahepatic, perihilar, and distal CCA. CCA is often diagnosed at an advanced stage due to the lack of early specific symptoms, resulting in poor prognosis and limited treatment options. Early detection remains critical for improving survival outcomes. Surgical resection represents the optimal therapeutic approach for achieving long-term survival, yet merely 20–30% of patients are candidates for surgery. Chemotherapy is commonly used in cases where surgery is not feasible. Nonetheless, the chemotherapy of CCA is challenged by drug resistance driven by tumor heterogeneity and the occurrence of severe systemic side effects.^1^ In recent years, immunotherapies and targeted therapies have emerged as promising treatment options.^2^ Despite advancements in treatment, the prognosis of CCA remains dismal, with 5-year overall survival between 7 and 20%.^3^ Continued research to develop highly effective treatments for patients with advanced CCA is essential.

Cantharidin is a natural terpenoid compound secreted primarily from beetles of the genus *Mylabris*.^4^ Cantharidin is a potent cytotoxin that effectively suppresses the growth and proliferation of various cancer cells. It inhibits DNA and RNA synthesis, triggers apoptosis, impacts immune responses, and effectively suppresses a wide range of cancer cell lines.^5^ Although cantharidin exhibits certain toxic effects on the human body, its anticancer potential remains undeniable.^6^ Cantharidin has demonstrated effectiveness against primary liver cancer,^7^ pancreatic cancer,^8^ gastric cancer,^9^ colorectal cancer,^10^ breast cancer,^11^ and lung cancer.^12^ Cantharidin is also effective against CCA. Previous research on the antitumor effects of cantharidin primarily focused on cell lines that do not represent the tissue specificity and biological characteristics of primary tumors. Consequently, developing a more clinically predictive *in vitro* model is essential to accurately assess cantharidin’s antitumor activity and elucidate its mechanism of action.

Patient-derived organoids (PDOs) are three-dimensional structures derived from patient tumor tissues, induced by adding specific extracellular matrix analogues and cytokines, closely resembling the architecture of primary tumors.^13^ Unlike traditional cell culture, PDOs overcome the limitations of two-dimensional systems, better replicating the physiological functions and characteristics of primary tumor tissues. Compared with animal models, PDOs reduce experimental costs and complexity, enhance construction efficiency, and offer other advantages such as rapid expansion, long-term cultivation, and real-time imaging *in vitro*, making PDOs excellent models for preclinical drug screening.^14^^,^^15^

In this study, we established CCA PDOs using surgically resected tumor specimens and demonstrated the homology between the PDOs and the original tissues through histopathology and genomic sequencing. We further evaluated the anticancer activity of cantharidin across multiple CCA PDO models, with comparative analysis against standard chemotherapy regimens. To elucidate the molecular basis of cantharidin’s antitumor activity in CCA, we conducted integrated transcriptome profiling and network pharmacology results, further validating these findings in patient-derived organoids-based xenograft (PDOX) mice.

## Results

### Construction and histopathological features of CCA PDOs

**Figure 1:**
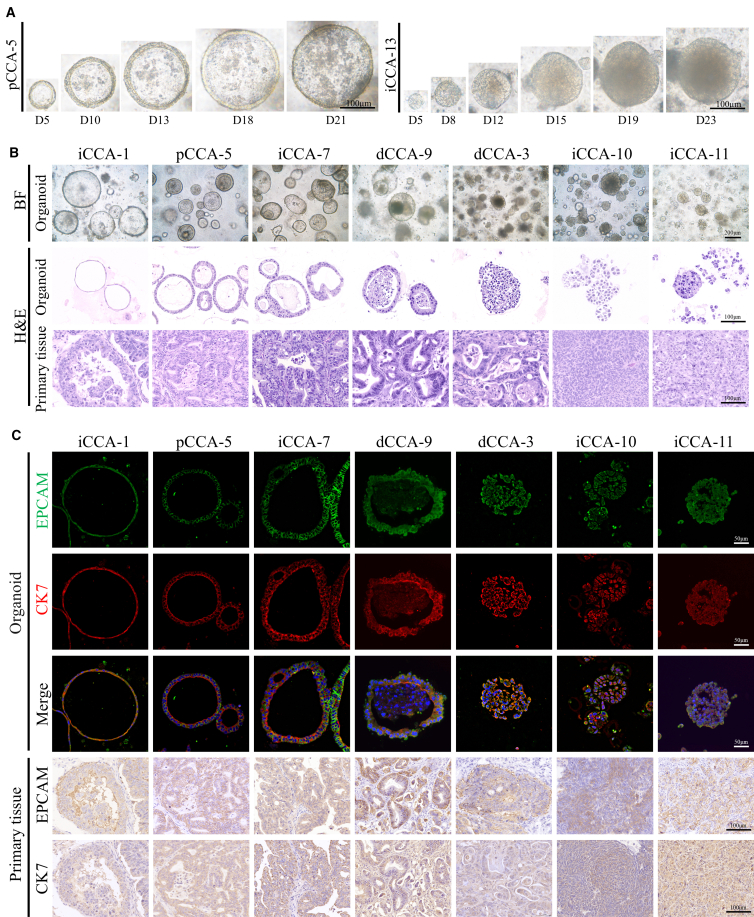
Establishment of CCA PDOs and histological characterization (A) Time course of CCA PDO culture exhibiting distinct morphologies: pCCA-5 (spherical cystic structures) and iCCA-13 (dense solid structures). (B) Bright field and H&E comparison of 7 CCA PDOs with the corresponding tumors. (C) IF staining of CCA PDOs and IHC staining of CCA primary tumor tissues for the bile duct markers (EPCAM and CK7).

In this study, we successfully established 16 CCA PDOs from 25 CCA patients who underwent surgical resection, achieving a success rate of 64%. These PDOs were stably maintained for over 10 passages, and drug sensitivity testing was performed at approximately passage 5 after cryopreservation at passages 3–5. Patient baseline characteristics are provided in Table S1. During culture, small cell masses emerged around day 5 and showed substantial growth in size and cellularity within 10–14 days (Figure 1A). PDOs derived from the same patient were morphologically similar. Bright-field and hematoxylin and eosin (H&E) staining identified two distinct morphological types in CCA PDOs: typical spherical cystic structures (with or without internal cells) and dense solid structures (Figure 1B). Immunohistochemistry (IHC) and immunofluorescence (IF) staining for cholangiocyte markers (EPCAM and CK7) confirmed strong expression in both PDOs and primary tumor tissues, indicating that PDOs retain key biological features of the original tumors (Figure 1C).

### Genomic characterizations of CCA PDOs

**Figure 2:**
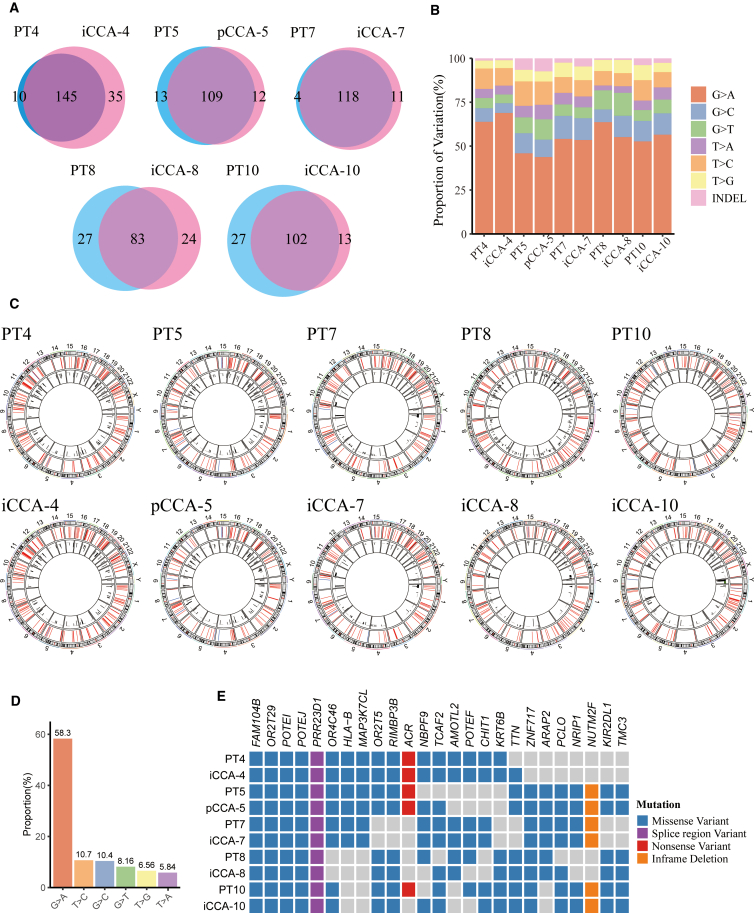
Genomic characteristics in paired tissue/PDO samples (A) The Venn diagrams illustrate the number of cancer-associated non-synonymous mutations in paired tissue/PDO samples. (B) Percentage of the 6 single-nucleotide variants (SNVs) and insertions and deletions (Indels) averaged across all samples. (C) The Circos plot shows that track 1 displays all SNVs (in red) and Indels (in blue) across all samples, and track 2 shows all copy number variations across all samples. (D) The proportions of the 6 types of SNVs and Indels across the samples. (E) Distribution map of the top 25 cancer-associated non-synonymous mutations across all samples. PT, primary tissue.

Consistent with published findings,^16^^,^^17^ our data confirm that PDO models maintain the characteristic mutational signatures of their parental tumor specimens. This study conducted whole-exome sequencing on iCCA-4, pCCA-5, iCCA-7, iCCA-8, and iCCA-10, along with their respective primary tumor samples (PT4, 5, 7, 8, and 10). The sequencing results revealed that primary tumors and corresponding PDOs contained approximately 100 tumor-associated non-synonymous mutations (Figure 2A; Table S2). Analysis of single-nucleotide variants (SNVs) and insertions and deletions (Indels) further revealed that the overall mutational profile of PDOs closely mirrored that of the primary tumor tissue (Figure 2B), with G>A mutations being the most prominent variant type (Figure 2D). Beyond SNVs and Indels, these PDOs also exhibited a high degree of similarity to their corresponding primary tumors in terms of copy number variations (Figure 2C). Among the top 25 most frequently mutated genes, PDOs exhibited a high level of concordance with their corresponding primary tumors (Figure 2E).

### Cantharidin drug screening results were compared with gemcitabine, cisplatin, and Adriamycin

**Figure 3:**
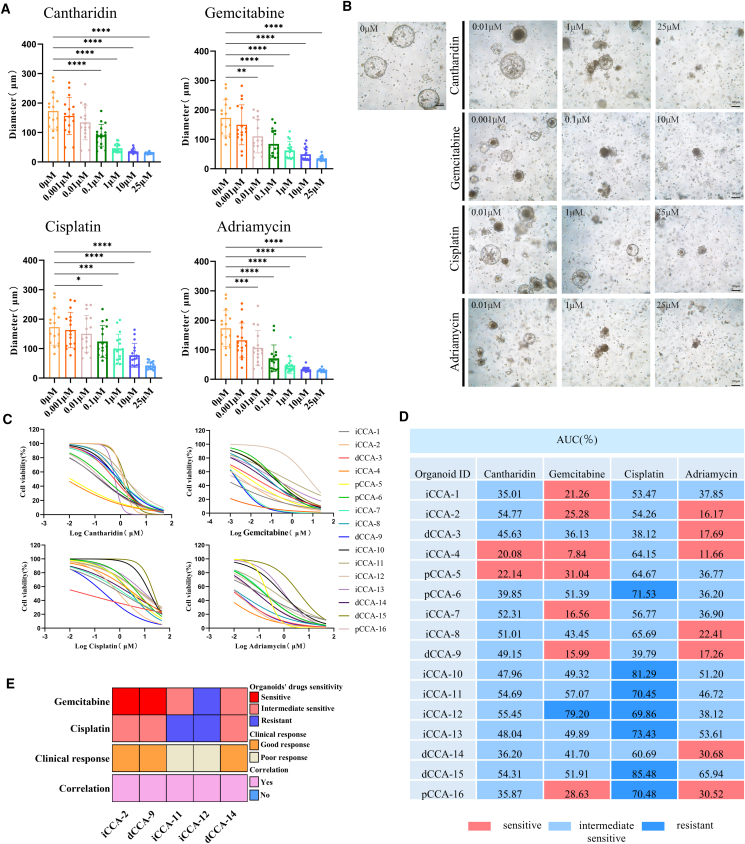
Cantharidin drug sensitivity results and comparison with gemcitabine, cisplatin, and Adriamycin (A) The diameter changes of 16 CCA PDOs for the different treatment groups. (B) Representative images of iCCA-8 treated with different concentrations of cantharidin, gemcitabine, cisplatin, and Adriamycin. (C) Dose-response curves generated from 16 CCA PDOs treated with different concentrations of cantharidin, gemcitabine, cisplatin, and Adriamycin. (D) Normalized area under the curve (AUC)% values for the different treatment groups of 16 CCA PDOs. Sensitive (lowest 33% AUC), resistant (highest 33% AUC), and intermediate response (middle 34% AUC). (E) The heatmap summarizes the clinical response of 5 CCA PDOs and corresponding patient drug responses. Data are presented as mean ± standard deviation (SD) (*n* = 3). ∗*p* < 0.05, ∗∗*p* < 0.01, ∗∗∗*p* < 0.001, and ∗∗∗∗*p* < 0.0001. One-way ANOVA was used to compare between different groups.

We evaluated the antitumor efficacy of cantharidin against CCA PDOs in comparison with gemcitabine, cisplatin, and Adriamycin. All four agents reduced PDO diameter in a dose-dependent manner. At 0.01 μM, gemcitabine and Adriamycin significantly decreased PDO diameters relative to controls, whereas cantharidin and cisplatin required 0.1 μM to achieve a significant effect (Figures 3A and 3B; Table S3).

Cell viability assays quantified live cells in the 16 PDOs post treatment, enabling the generation of dose-response curves and calculating IC50 values. The IC50 values of cantharidin ranged from 7.06 nM to 1.65 μM, with an average value of 0.71 μM. In comparison, gemcitabine displayed IC50 values from 0.01 nM to 6.25 μM, with an average of 0.47 μM. For cisplatin, IC50 values ranged from 35.48 nM to 23.11 μM, with an average of 5.94 μM, while Adriamycin showed IC50 values ranging from 3.17 nM to 4.18 μM, with an average of 0.48 μM (Figure 3C; Table S4). These findings indicate that cantharidin exhibits stronger antitumor activity than cisplatin but slightly less potency than gemcitabine and Adriamycin.

The area under the curve percent values of cantharidin were further calculated to evaluate its sensitivity across the 16 PDOs. Differences in response were observed, with iCCA-4 and pCCA-5 showing marked sensitivity to cantharidin, while the remaining PDOs exhibited intermediate sensitivity. Sensitivity patterns to the other agents also varied: iCCA-1, iCCA-2, iCCA-4, pCCA-5, iCCA-7, dCCA-9, and pCCA-16 were sensitive to gemcitabine, with iCCA-12 classified as resistant, while the others were intermediate sensitive. pCCA-6, iCCA-10, iCCA-11, iCCA-12, iCCA-13, dCCA-15, and pCCA-16 were resistant to cisplatin, while the remaining PDOs exhibited intermediate sensitivity. For Adriamycin, iCCA-2, dCCA-3, iCCA-4, iCCA-8, dCCA-9, dCCA-14, and pCCA-16 were classified as sensitive, and the other PDOs showed intermediate sensitivity (Figure 3D).

Previous studies have indicated that drug screening results using PDOs correlate with patients' clinical responses to chemotherapy.^18^ We collected clinical data from five CCA patients who received adjuvant chemotherapy after surgery (Table S5). According to the BILCAP trial criteria,^19^ recurrence within 17.5 months after surgery is considered indicative of a poor clinical response to chemotherapy. Among these patients, iCCA-2, dCCA-9, and dCCA-14 were classified as chemotherapy-sensitive responders, while iCCA-11 and iCCA-12 were classified as chemotherapy-resistant responders (Figure 3E). In the drug screening assays using CCA PDOs, a chemotherapy regimen was defined as resistant if it included any chemotherapeutic drug to which resistance was observed.^20^ All five cases showed consistency between the drug sensitivity results of PDOs and the clinical responses of the corresponding patients.

To preliminarily assess cantharidin safety, we generated normal cholangiocyte organoids from three individuals. H&E and IHC staining for EPCAM and CK7 confirmed their identity (Figure S1A). Treatment with 1 μM cantharidin led to a mild volume reduction in normal organoids but preserved structural integrity (Figure S1B). In contrast, CCA PDOs showed marked shrinkage and fragmentation (Figure 3B), indicating selective cantharidin cytotoxicity toward CCA cells and supporting its potential clinical safety.

### Transcriptomic analysis of CCA PDOs upon 0.5 μM cantharidin

**Figure 4:**
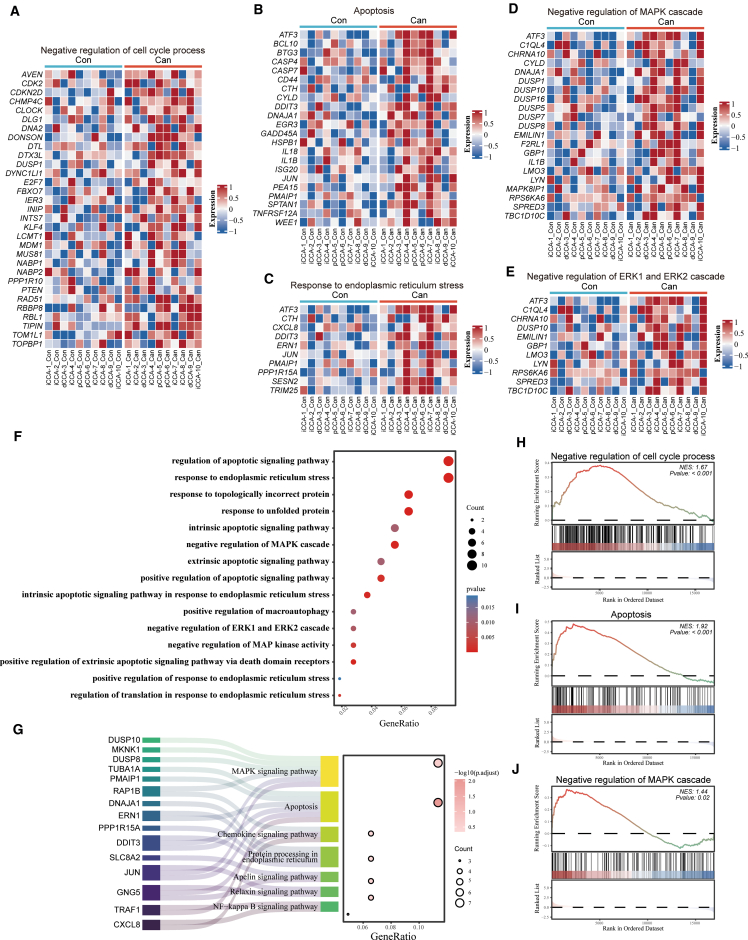
Transcriptome analysis of 0.5 μM cantharidin-treated CCA PDOs (A–E) The Heatmap reveals the expression of genes related to the negative regulation of cell cycle process (A), apoptosis (B), response to endoplasmic reticulum stress (C), the negative regulation of MAPK cascade (D), and the negative regulation of ERK1/2 cascade (E). (F and G) GO (F) and KEGG (G) enrichment analysis of the differentially expressed genes (DEGs) upregulated in cantharidin-treated CCA PDOs. (H–J) GSEA related to the negative regulation of cell cycle process (H), apoptosis (I), and the negative regulation of MAPK cascade (J). Con, control; Can, cantharidin.

To investigate cantharidin’s mechanism of action, we performed transcriptomic analysis on 10 CCA PDOs treated with 0.5 μM cantharidin. Heatmaps showed upregulation of genes involved in cell cycle negative regulation and apoptosis (Figures 4A and 4B), as well as endoplasmic reticulum stress response (Figure 4C). Extensive evidence has established that the mitogen-activated protein kinase (MAPK) signaling axis has been closely linked to CCA progression through its modulation of fundamental biological processes such as cell cycle progression, apoptotic resistance, and neovascularization.^21^^,^^22^^,^^23^ The heatmap showed that the MAPK cascade was significantly downregulated in the cantharidin-treated CCA PDOs (Figure 4D). The ERK1/2 pathway is one of the main branches of the MAPK signaling axis. Within the MAPK pathway, cantharidin upregulated negative regulators ATF3, DUSP10, and SPRED3, inhibiting ERK1/2 phosphorylation (Figure 4E). Gene ontology (GO) enrichment analysis highlighted apoptosis, endoplasmic reticulum stress response, and MAPK downregulation (Figure 4F). Kyoto Encyclopedia of Genes and Genomes (KEGG) analysis revealed enrichment in MAPK signaling, apoptosis, and endoplasmic reticulum protein processing pathways (Figure 4G). Gene Set Enrichment Analysis (GSEA) confirmed downregulation of cell cycle and MAPK cascade and enhanced apoptosis (Figures 4H–4J). These findings indicate that cantharidin predominantly induces cell-cycle arrest, apoptosis, endoplasmic reticulum stress, and MAPK pathway suppression.

We identified the intersection between the mutated genes in the five PDOs and the differentially expressed genes following cantharidin treatment, which yielded six overlapping genes: ZFPL1, STK11IP, ALKBH3, PDE6C, MUS81, and SPTBN5 (Figure S2A). However, these genes were not associated with cantharidin sensitivity (iCCA-4 and pCCA-5 are sensitive to cantharidin) (Figure S2B). Additionally, we found that all five PDOs shared mutations in FAM104B, OR2T29, POTEI, POTEJ, PRR23D1, and TCAF2. Mutations in ABCA13, ACR, KIAA1522, and NPIPB2 were observed in cantharidin-sensitive iCCA-4 and pCCA-5; however, due to the limited sample size of cantharidin-sensitive PDOs, these findings lack representativeness.

### Network pharmacology analysis of cantharidin for the treatment of CCA

**Figure 5:**
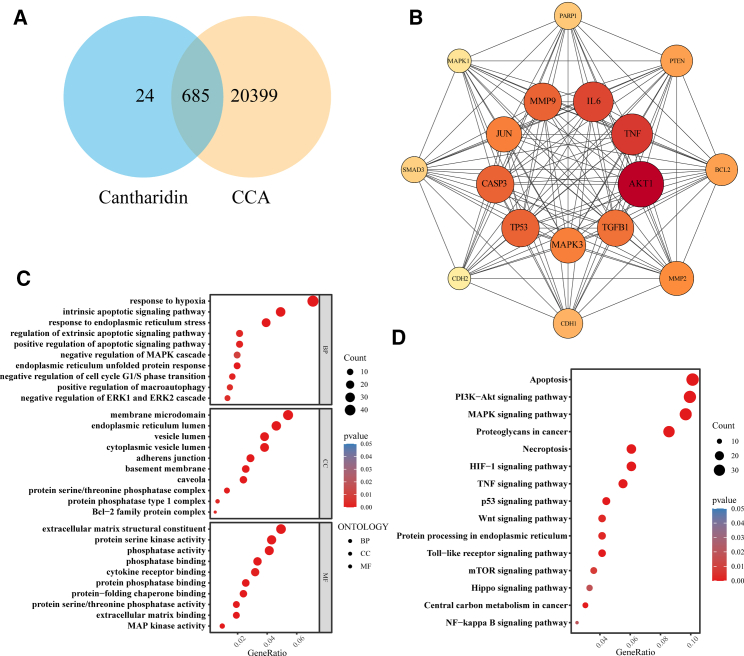
Network pharmacology analysis of cantharidin for treatment of CCA (A) Venn diagram of cantharidin and CCA targets. (B) PP-I network diagram. (C) GO enrichment analysis diagram. (D) KEGG enrichment analysis diagram.

A total of 709 targets of cantharidin were obtained from the TCMSP, HERB, CTD, and Swiss Target Prediction databases. Using the GeneCards, CTD, and TTD databases, targets with a Relevance Score ≥1 for CCA were selected, resulting in 21,084 CCA targets after merging and deduplication. The intersection of these datasets yielded 685 common targets (Figure 5A). Using the CytoNCA plugin, core targets were screened based on six centrality measures (Betweenness, Closeness, Degree, Eigenvector Centrality, Network Centrality, and Local Average Connectivity-based Method), with values above the median. After three rounds of screening, 17 core targets, including MAPK1(ERK2) and MAPK3(ERK1), were identified (Figure 5B). GO enrichment analysis indicated that targets of cantharidin and CCA were primarily involved in response to hypoxia, response to endoplasmic reticulum stress, positive regulation of apoptotic signaling pathway, and negative regulation of MAPK cascade (Figure 5C). KEGG enrichment analysis indicated that these targets were mainly enriched in apoptosis, PI3K-Akt signaling pathway, and MAPK signaling pathway (Figure 5D).

### Cantharidin induces apoptosis and inhibits proliferation by downregulating p-ERK1/2-c-Fos signaling pathway

**Figure 6:**
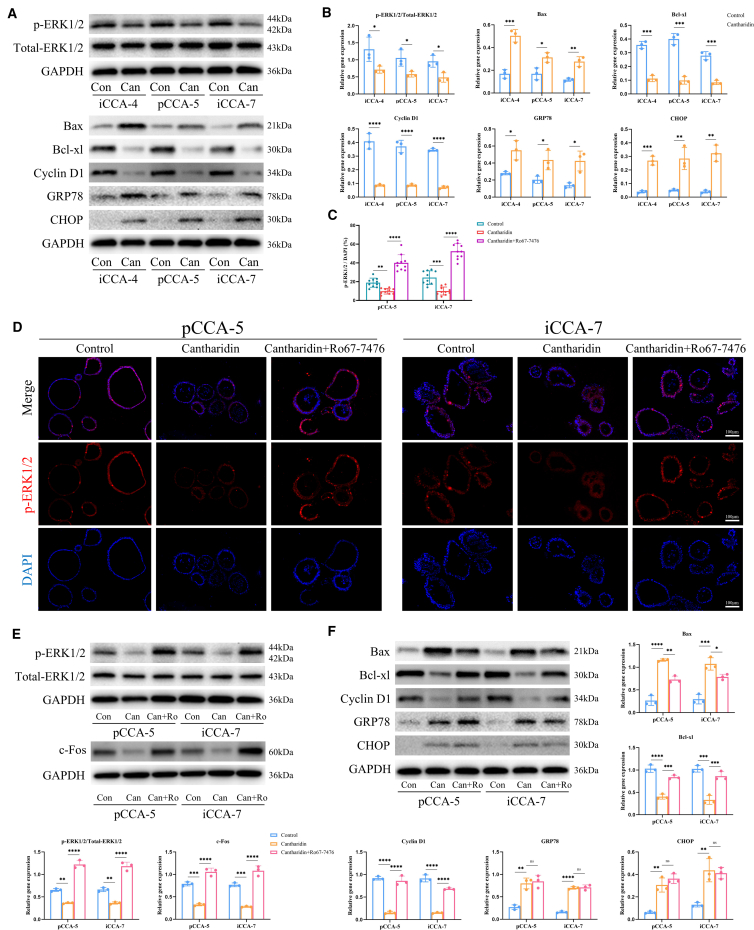
Cantharidin induces apoptosis and inhibits proliferation by downregulating p-ERK1/2–c-Fos signaling pathway (A) Western blot (WB) analysis and corresponding quantification (B) of p-ERK1/2, apoptosis-related proteins (Bax and Bcl-xL), a marker of cell cycle progression (Cyclin D1), and endoplasmic reticulum stress response markers (GRP78 and CHOP). (*n* = 3). (C and D) Quantification (C) of p-ERK1/2 staining and representative IF images (D) (*n* = 10). (E) WB analysis and quantification of p-ERK1/2 and c-Fos (*n* = 3). (F) WB analysis and quantification of Bax, Bcl-Xl, Cyclin D1, GRP78, and CHOP (*n* = 3). Con, control; Can, cantharidin; Can+Ro, Cantharidin+Ro67-7476. Data are presented as mean ± SD. ∗*p* < 0.05, ∗∗*p* < 0.01, ∗∗∗*p* < 0.001, and ∗∗∗∗*p* < 0.0001, ns: no significant difference. t test was used to compare between two groups. One-way ANOVA was used to compare between three groups.

Western blot experiments were conducted to assess the protein levels associated with the p-ERK1/2, apoptosis, cell cycle, and endoplasmic reticulum stress in iCCA-4, pCCA-5, and iCCA-7 following cantharidin treatment. After treatment with cantharidin, the levels of p-ERK1/2 were downregulated in all three PDOs. Additionally, the pro-apoptotic protein Bax was significantly upregulated, while the anti-apoptotic protein Bcl-xl was significantly reduced in iCCA-4, pCCA-5, and iCCA-7 following cantharidin treatment. Cyclin D1 levels were decreased, and endoplasmic reticulum stress proteins GRP78 and CHOP were significantly elevated in iCCA-4, pCCA-5, and iCCA-7 following cantharidin treatment (Figures 6A and 6B), consistent with the transcriptomic sequencing data and network pharmacology analysis.

The p-ERK1/2 staining revealed a reduction in the nuclear translocation of p-ERK1/2 in pCCA-5 and i-CCA7 after cantharidin treatment. Ro67-7476 is a potent agonist of p-ERK1/2.^24^^,^^25^^,^^26^^,^^27^ Notably, the administration of Ro67-7476 before cantharidin treatment effectively reversed the p-ERK1/2 negative regulatory and subsequently upregulated c-Fos expression (Figures 6C–6E).

The activation of p-ERK1/2 resulted in the downregulated expression of Bax and the upregulated expression of Bcl-xl and Cyclin D1 in pCCA-5 and iCCA-7. However, the expression levels of endoplasmic reticulum stress markers (GRP78 and CHOP) were not significantly altered (Figure 6F). These findings indicate that cantharidin inhibits proliferation and induces apoptosis in CCA PDOs by downregulating p-ERK1/2-c-Fos signaling pathway, but the endoplasmic reticulum stress cantharidin promotes is not sufficient to trigger cell apoptosis.

### Cantharidin’s antitumor effects in pCCA-5 and iCCA-7 were reversed by Ro67-7476

**Figure 7:**
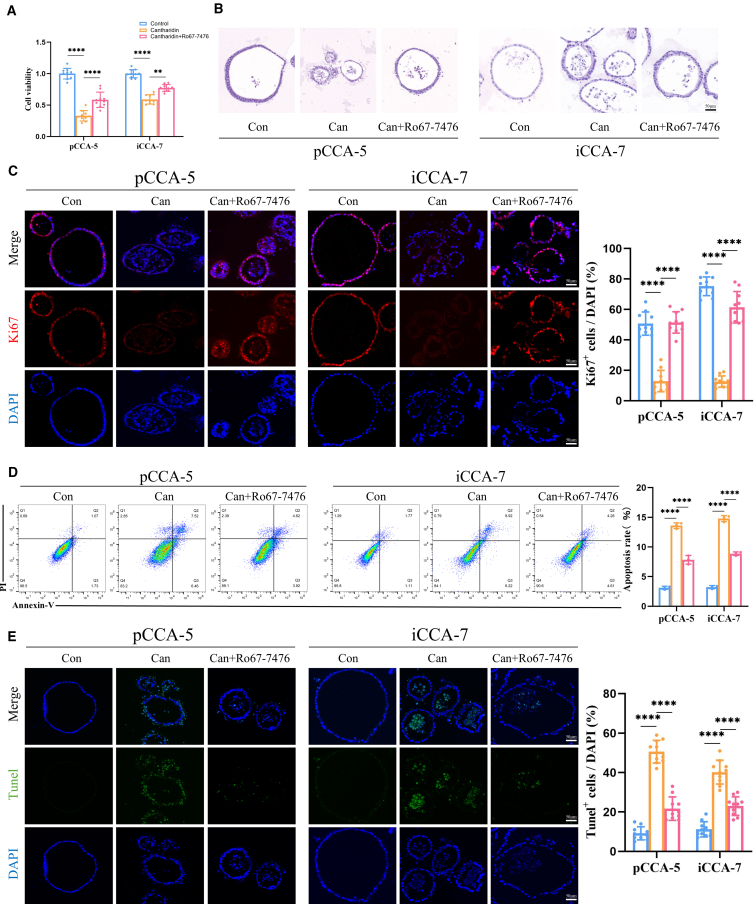
Ro67-7476 reversed the antitumor effects induced by cantharidin in pCCA-5 and iCCA-7 Cell viability (A) (*n* = 10) and H&E stain (B) in the control group, cantharidin-treated group, and cantharidin combined with the Ro67-7476 co-treatment group in pCCA-5 and iCCA-7. (C) Representative Ki67 staining images of pCCA-5 and iCCA-7 with different treatments and quantitative analysis of Ki67^+^ cells (*n* = 10). (D) Apoptosis detection in pCCA-5 and iCCA-7 via flow cytometry (*n* = 3). (E) Representative TUNEL staining images of pCCA-5 and iCCA-7 with different treatments and quantitative analysis of TUNEL^+^ cells (*n* = 10). Con, control; Can, cantharidin. The data are presented as mean ± SD. ∗∗*p* < 0.01, ∗∗∗∗*p* < 0.0001. One-way ANOVA was used to compare between different groups.

We demonstrated that Ro67-7476 can alleviate the reduction of cell cycle proteins Cyclin D1 caused by cantharidin by activating p-ERK1/2. Ro67-7476 pretreatment substantially enhanced PDO cell viability and resisted the antitumor effects induced by cantharidin (Figure 7A). The H&E results revealed that PDOs in the cantharidin group showed more signs of structural disintegration compared with those in the cantharidin + Ro67-7476 group (Figure 7B). Additionally, Ki67-positive cells significantly increased in the cantharidin + Ro67-7476 group compared with the cantharidin group (Figure 7C). The Apoptosis assay reveals a lower apoptosis level in the cantharidin + Ro67-7476 group compared with the cantharidin group (Figures 7D and 7E). Overall, cantharidin’s antitumor effects by downregulating p-ERK1/2 expression in CCA PDOs were reversed by Ro67-7476.

### Cantharidin pharmacokinetic assessment in PDOXs

**Figure 8:**
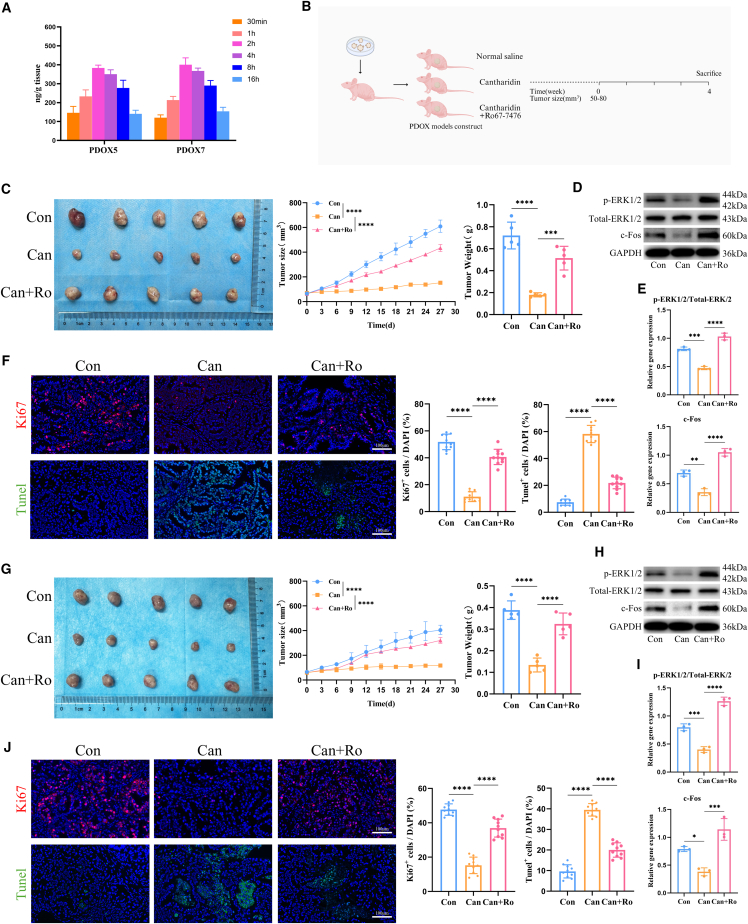
Cantharidin exerts antitumor effects by downregulating the p-ERK1/2-c-Fos signaling pathway in PDOXs (A) Intratumoral concentrations of cantharidin in PDOX5 and PDOX7 models following a single oral administration at a dose of 1 mg/kg (*n* = 5). (B) Schematic of the pharmacodynamic experiments. (C) Representative image, growth curves, and tumor weight of PDOX5 after administration for 4 weeks (*n* = 5). (D) Expression levels and corresponding quantification (E) of p-ERK1/2 and c-Fos of PDOX5 after administration for 4 weeks (*n* = 3). (F) Representative Ki67 and TUNEL staining images of PDOX5 with different treatments and quantitative analysis of Ki67^+^ and TUNEL^+^ cells (*n* = 10). (G) Representative image, growth curves, and tumor weight of PDOX7 after administration for 4 weeks (*n* = 5). (H) Expression levels and corresponding quantification (I) of p-ERK1/2 and c-Fos of PDOX7 after administration for 4 weeks (*n* = 3). (J) Representative Ki67 and TUNEL staining images of PDOX7 with different treatments and quantitative analysis of Ki67^+^ and TUNEL^+^ cells (*n* = 10). Con, control; Can, cantharidin; Can+Ro, Cantharidin+Ro67-7476. The data are presented as mean ± SD. ∗*p* < 0.05, ∗∗*p* < 0.01, ∗∗∗*p* < 0.001, ∗∗∗∗*p* < 0.0001. One-way ANOVA was used to compare between different groups.

Intratumoral concentrations of cantharidin in PDOX5 and PDOX7 models were shown in Figure 8A. The results demonstrate that the intratumoral concentration of cantharidin peaked at approximately 2 h post administration and subsequently declined in a time-dependent manner. A therapeutically relevant concentration (around 300 ng/g) was maintained for up to 6 h, providing a crucial kinetic profile for its antitumor effects.

### Cantharidin exerts antitumor effects by downregulating the p-ERK1/2-c-Fos signaling pathway *in vivo*

Given that Ro67-7476 has been shown to counteract the antitumor effects of cantharidin *in vitro*, we further evaluated the efficacy of Ro67-7476 *in vivo* (Figure 8B). Mice engrafted with pCCA-5 and iCCA-7 demonstrated a significant response to cantharidin treatment, evidenced by substantial reductions in both tumor growth and tumor weight compared with untreated controls. Nevertheless, the PDOX5 and PDOX7 mice exhibited greater tumor growth and tumor weight in the cantharidin + Ro67-7476 group compared with the cantharidin group (Figures 8C and 8G). In PDOX5 and PDOX7 mice, Ro67-7476 effectively reversed the cantharidin-induced suppression of p-ERK1/2 and subsequently upregulated c-Fos expression (Figures 8D, 8E, 8H, and 8I). Additionally, apoptosis and proliferation within PDOX tissues were assessed using TUNEL staining for apoptosis and Ki67 staining for proliferation. The results revealed that there were fewer Ki67^+^ cells and more TUNEL^+^ cells in the cantharidin group than in the cantharidin + Ro67-7476 group in PDOX5 and PDOX7 (Figures 8F and 8J). These results suggested that cantharidin exerts antitumor effects by downregulating the p-ERK1/2-c-Fos signaling pathway.

## Discussion

CCA exhibits limited sensitivity to commonly used clinical chemotherapeutic agents. Therefore, developing more effective therapeutic drugs for CCA is crucial to enhancing treatment efficacy and improving patient prognosis. Compared with conventional drugs, certain herbal compounds have been recognized for their antitumor properties and lower incidence of side effects for CCA, offering notable advantages.^18^ Cantharidin, a potent cytotoxic compound, has been proven to effectively inhibit the proliferation of various tumor cell types, exhibiting significant anticancer activity. As a promising antitumor drug, cantharidin has been clinically applied as an adjuvant therapy for multiple types of gastrointestinal malignancies. For example, cantharidin effectively inhibits the growth of pancreatic cancer cell lines, leading to G2/M cell-cycle arrest, inducing apoptosis through both extrinsic and intrinsic pathways, and suppressing the invasive ability of pancreatic cancer cells, while exhibiting much lower toxicity to normal pancreatic duct cells.^28^^,^^29^ Song et al. found that cantharidin inhibited the growth, migration, and invasion of gastric cancer cells by downregulating the expression of CCAT1 and suppressing the activation of the PI3K/Akt signaling pathway.^9^ In addition, cantharidin can significantly inhibit the growth and migration of hepatocellular carcinoma and colorectal cancer cell lines.^10^^,^^30^ However, the research on cantharidin in CCA remains limited, with most studies focusing primarily on tumor cell lines. Therefore, further investigations are necessary to explore the antitumor effects of cantharidin in CCA, using more precise and sensitive *in vitro* models that more accurately reflect individual patient variations.

PDOs largely maintain the histological features and genomic alterations of their corresponding primary tumors, making them suitable for translational research and personalized medicine.^31^^,^^32^ Since the establishment of the primary liver cancer PDO in 2017 for drug screening, PDOs have been widely used in drug development and in guiding clinical treatment.^33^ Lee et al. classified ICC PDOs into small duct (SD) and large duct types based on histological features. Drug response testing revealed that SD-type PDOs were more sensitive to gemcitabine and cisplatin and had a better prognosis. Further analysis of genomic sequencing results identified potential specific targeted pathways for different types of ICC patients, paving the way for personalized treatment.^34^ Besides, Li et al. established 27 ICC PDOs and tested them with 129 anticancer drugs, revealing significant heterogeneity in patient responses to these drugs.^35^ Saito et al. performed a high-throughput drug screening of CCA PDOs using 339 clinical drugs and found that the antifungal agents amorolfine and fenticonazole inhibited the growth of CCA PDOs with minimal toxicity to normal biliary epithelial cells.^36^ Additionally, Xiaoxue et al. established 61 CCA PDOs and conducted drug screening using gemcitabine, cisplatin, 5-fluorouracil, and oxaliplatin. The drug screening results were validated in 92.3% (12/13) of CCA patients with actual clinical responses.^18^ Jianhua et al. also guided the postoperative medication of an ICC patient based on the results of PDO drug screening, leading to good clinical efficacy.^37^ These findings highlight the crucial role of PDOs in enabling personalized drug therapy for CCA patients.

In this study, 16 CCA PDOs were successfully established from tumor tissues of CCA patients, and their histopathological and genomic characteristics closely matched those of the primary tumors. In general, the morphology of PDOs typically exhibits two distinct forms: a typical ring-like structure or a solid structure. Additionally, the expression patterns of cholangiocyte-related markers, such as EPCAM and CK7, in the PDOs were consistent with those in the primary tumors. Given that few CCA patients benefit from genetic testing, such as next-generation sequencing, PDOs present a promising preclinical tool for drug testing and personalized treatment of CCA.

The sensitivity of 16 CCA PDOs to cantharidin was assessed. The diameters of the PDOs decreased with increasing concentrations of cantharidin, eventually collapsing, demonstrating a clear dose-dependent effect. Gemcitabine combined with cisplatin is a commonly used chemotherapy regimen for CCA and has shown good efficacy in the treatment of advanced CCA.^38^ Adriamycin is a broad-spectrum chemotherapeutic agent that acts on various tumors and is often used in localized chemotherapy for CCA.^39^ We compared the antitumor effects of cantharidin with gemcitabine, cisplatin, and Adriamycin on the 16 PDOs, finding that cantharidin exhibited stronger antitumor activity than cisplatin, but its efficacy was slightly lower than that of gemcitabine and Adriamycin. However, the myelosuppression induced by gemcitabine and the cardiotoxicity induced by Adriamycin highlight the need for further exploration of safer drugs with fewer side effects for CCA treatment. Based on the results of this study, cantharidin has potential in CCA clinical treatment as a supplement to gemcitabine and Adriamycin or as a substitute for cisplatin. In combination therapy, cantharidin may act synergistically with conventional chemotherapy agents (e.g., gemcitabine, cisplatin) or molecularly targeted drugs, particularly those modulating overlapping apoptotic and protein synthesis pathways.

Despite its beneficial anticancer properties, the clinical translation of cantharidin is challenged by its associated toxicities, including nephrotoxicity, hepatotoxicity, hematochezia, and tenesmus.^40^^,^^41^ To mitigate its systemic toxicity, several strategies have been explored. Ethanolamine has been identified as a potent antidote that specifically rescues cantharidin-induced cytotoxicity by modulating phosphatidylethanolamine-related functions.^42^ Moreover, numerous analogues—such as norcantharidin, norcantharimide, cantharidinamides, sodium cantharidate, anhydride-modified derivatives, and N-hydroxycantharidimide—have been synthesized to improve its safety profile while retaining anticancer efficacy.^41^ Encapsulation strategies using liposomal or nanoemulsion formulations have also shown considerable potential in reducing systemic exposure and toxicity without compromising antitumor activity.^43^^,^^44^ These approaches provide a viable pathway for leveraging the therapeutic benefits of cantharidin in a clinically acceptable window. Besides, the concordance between the PDO drug screening results and the observed clinical outcomes in all five patients is a significant finding. It strongly validates the use of PDOs as a reliable *ex vivo* platform for functional precision oncology. This model holds immense potential to guide personalized treatment decisions by identifying effective regimens and, crucially, by preventing the administration of ineffective chemotherapy, thereby sparing patients from unnecessary toxicity. Future studies with larger patient cohorts are warranted to further solidify these findings and explore their applicability across diverse cancer types.

**Figure 9:**
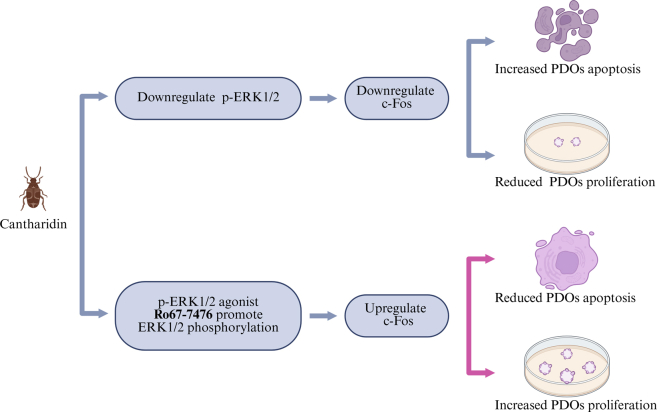
Mechanism diagram: cantharidin exerts antitumor effects by downregulating the p-ERK1/2-c-Fos signaling pathway.

The ERK1/2-c-Fos pathway has been described as a linchpin tumorigenic mechanism associated with CCA.^45^^,^^46^ Our findings demonstrate that cantharidin significantly inhibits the growth and viability of CCA PDOs by suppressing p-ERK1/2-c-Fos signaling pathway, leading to attenuated proliferation and enhanced apoptosis *in vivo* and *in vitro* (Figure 9). This finding reveals a distinct mechanism underlying cantharidin’s antitumor efficacy in CCA, which is distinct from but potentially interconnected with previously reported pathways. These findings invite an interesting comparison with the established work by Zhou et al., which demonstrated that cantharidin inhibits PP2A activity, resulting in nuclear factor κB activation and inhibition of cell migration and invasion.^47^ The observed suppression of p-ERK1/2-c-Fos signaling pathway in our study presents an apparent paradox, as PP2A inhibition would typically enhance ERK1/2 activation. This discrepancy may be attributed to differences in experimental models (cell lines vs. PDOs) or may reflect the multi-target nature of natural compounds like cantharidin, which can simultaneously engage several signaling nodes.

### Limitations of the study

We successfully established 16 CCA PDOs from 25 CCA patients. Utilizing these PDOs, we demonstrated that cantharidin exerts its antitumor effects through inhibition of the p-ERK1/2-c-Fos pathway. Nevertheless, this study still has several limitations. First, a major limitation in pharmacogenomic studies using PDOs is the risk of false discoveries due to small sample sizes. In this study, the limited number of cantharidin-sensitive PDOs (*n* = 2) means that mutations such as those in ABCA13, ACR, KIAA1522, and NPIPB2 may not be representative biomarkers of drug sensitivity. These findings should therefore be considered preliminary. Future validation in a larger cohort is essential to reliably link genetic variants to cantharidin response. Second, CCA PDOs lack the tumor microenvironment (TME), including immune cells, stromal components, and vascular networks, which are critical for studying immunotherapy and tumor-stroma interactions. This absence restricts a comprehensive assessment of cantharidin’s full therapeutic potential, including its possible immunomodulatory effects and on-target/off-tumor toxicities within a more complex physiological context. To bridge this gap, future work will focus on developing more sophisticated co-culture systems. We plan to integrate immune cells, such as patient-derived tumor-infiltrating lymphocytes or macrophages, and cancer-associated fibroblasts with our PDOs. Techniques such as microfluidic-based organ-on-a-chip platforms^48^^,^^49^^,^^50^ will be explored to better mimic the dynamic interactions and spatial organization of the human TME. These advanced models will be crucial for validating the efficacy and safety of cantharidin and for elucidating its multifaceted mechanisms of action, ultimately accelerating its translational potential into more effective combination therapies. Third, long-term culturing may lead to genetic drift or clonal selection, altering the original tumor’s molecular characteristics. To mitigate this risk, we recommend conducting drug screening assays around passage 5 to ensure the preservation of genomic fidelity. Finally, the high costs and technical expertise required for CCA PDO generation and maintenance restrict their widespread use. This limitation could be mitigated through the development of standardized, cost-effective organoid culture protocols.

## Resource availability

### Lead contact

Further information and requests for resources and reagents should be directed to and will be fulfilled by the lead contact, Dr. Yamin Zhang (5020200824@nankai.edu.cn).

### Materials availability

All materials are available from the corresponding author on reasonable request.

### Data and code availability

• The raw data of RNA-seq datasets in this study have been deposited in the NCBI Sequence Read Archive, accession number: PRJNA1261625.
• This paper does not report original code.
• Any additional information for reanalyzing the data in this study is available from the lead contact upon request.

## Acknowledgments

This work was supported by the 10.13039/501100001809National Natural Science Foundation of China (82372194, 82204546), Tianjin Health Science and Technology Project (TJWJ2023QN034), Tianjin Science and Technology Project (24JCYBJC01840), State Key Laboratory of Druggability Evaluation and Systematic Translational Medicine project (712023001,712023002,712024001), Project of Science and Technology Program of Tianjin (24ZXZSSS00480, 24ZYCGSY00640), and CAMS Innovation Fund for Medical Sciences (2019-I2M-5-020).

## Author contributions

Conceptualization, P.H., L.Y., Z.W., and Y.Z.; methodology, P.H., L.Z., W.T., S.L., Z.L., X.Z., and T.Z.; software, L.W. and T.Y.; formal analysis & visualization, L.Z., T.W., and S.L.; data curation, L.W., T.Y., and W.T.; writing – original draft, P.H.; writing – review & editing, T.C., L.Y., Z.W., and Y.Z.; supervision & project administration, Z.W. and Y.Z.

## Declaration of interests

The authors declare no competing interests.

## STAR★Methods

### Key resources table

**Table:** 

REAGENT or RESOURCE	SOURCE	IDENTIFIER
**Antibodies**		

EPCAM	Proteintech	Cat# 66316-1-Ig; RRID: AB_2881697
CK7	Abcam	Cat# ab181598; RRID: AB_2783822
Phospho-ERK1/2	Affinity	Cat# AF1015; RRID: AB_2834432
ERK1/2	Proteintech	Cat# 11257-1-AP; RRID: AB_2139822
c-Fos	Proteintech	Cat# 66590-1-Ig; RRID: AB_2881950
Bax	Proteintech	Cat# 50599-2-Ig; RRID: AB_2061561
Bcl-xl	Proteintech	Cat# 26967-1-AP; RRID: AB_2880702
Cyclin D1	Proteintech	Cat# 60186-1-Ig; RRID: AB_10793718
GRP78	Proteintech	Cat# 11587-1-AP; RRID: AB_2119855
CHOP	Proteintech	Cat# 15204-1-AP; RRID: AB_2292610
GAPDH	Proteintech	Cat# 60004-1-Ig; RRID: AB_2107436
Ki67	Proteintech	Cat# 27309-1-AP; RRID: AB_2756525
Goat Anti-Mouse Secondary Antibody	Proteintech	Cat# RGAM001; RRID: AB_3068333
Goat Anti-Rabbit Secondary Antibody	Proteintech	Cat# RGAR001; RRID: AB_3073505
Goat Anti-Rabbit IgG H&L (Alexa Fluor® 594)	Abcam	Cat# ab150080; RRID: AB_2650602
Goat Anti-Mouse IgG H&L (Alexa Fluor® 488)	Abcam	Cat# ab150113; RRID: AB_2576208

**Biological samples**		

Human cholangiocarcinoma tissue	Tianjin First Central Hospital	Table S1

**Chemicals, peptides, and recombinant proteins**		

Advanced DMEM/F-12	Gibco	Cat# 12634010
Penicillin-streptomycin	Gibco	Cat# 15140122
GlutaMax	Gibco	Cat# 35050061
HEPES	Gibco	Cat# 15630106
B27 supplement without vitamin A	Gibco	Cat# A3353501
N2 supplement	Gibco	Cat# 17502048
N-acetyl-L-cysteine	MedChemExpress	Cat# HY-110256
Nicotinamide	MedChemExpress	Cat# HY-B0150
Gastrin I	MedChemExpress	Cat# HY-P1097
Forskolin	MedChemExpress	Cat# HY-15371
A83-01	MedChemExpress	Cat# HY-10432
Y-27632	MedChemExpress	Cat# HY-10071
Recombinant human EGF	Novoprotein	Cat# C029
Recombinant human FGF10	Novoprotein	Cat# CR11
Recombinant human HGF	Novoprotein	Cat# CJ72
Recombinant Human Noggin	Novoprotein	Cat# CB89
Recombinant Human R-spondin 1	Novoprotein	Cat# CX83
Recombinant Human Wnt3a	Novoprotein	Cat# C22R
Cantharidin	MedChemExpress	Cat# HY-N0209
Ro67-7476	MedChemExpress	Cat# HY-100403
Collagenase Type IV	Solarbio	Cat# C8160
Matrigel	Corning	Cat# 356231
TrypLE™ Express enzyme	Gibco	Cat# 12604021
Gemcitabine	MedChemExpress	Cat# HY-17026
Cisplatin	MedChemExpress	Cat# HY-17394
Adriamycin	Solarbio	Cat# ID8880
RIPA	Solarbio	Cat# R0010
PMSF	Solarbio	Cat# R0010

**Critical commercial assays**		

CellTiter-Glo 3D Reagent	Promega	Cat# G9682
BCA Protein Assay Kit	Solarbio	Cat# PC0020
Super ECL substrate	Elabscience	Cat# E-IR-R308
One-step TUNEL *In Situ* Apoptosis Kit	Elabscience	Cat# E-CK-A320
Annexin V-FITC/PI Apoptosis Detection Kit	MedChemExpress	Cat# HY-K1073

**Deposited data**		

RNA-Sequencing Data	This paper	PRJNA1261625

**Experimental models: organisms/strains**		

Male BALB/c-nu mice (6 weeks old)	Beijing Vital River Laboratory Animal Technology Co., Ltd	Cat# 401

**Software and algorithms**		

GraphPad Prism software	GraphPad 9.0	https://www.graphpad.com/
ImageJ	Open source	https://imagej.net/software/fiji/
FlowJo_v10.8.1	BD Biosciences	https://www.flowjo.com/

### Experimental model and study participant details

#### Patient sample acquisition

Tumor samples (*n* = 25) were collected from patients undergoing surgical resection in Tianjin First Central Hospital and confirmed as CCA tissue by pathology. This study was approved by the Ethics Committee of Tianjin First Central Hospital (Ethics Approval Number: 2020N221KY), and all patients participating in the study signed informed consent forms.

#### PDOX models establishment and pharmacokinetic assessment

Male BALB/c-nu mice (6 weeks old) were obtained from Beijing Vital River Laboratory Animal Technology Co., Ltd (Beijing, China). For the establishment of PDOX models, digested pCCA-5 and iCCA-7 cells were suspended in 100 μL of Matrigel at a density of 3×10^6^ cells per mouse, followed by subcutaneous injection into the right dorsal flank of each animal. Tumor growth was assessed every three days using a tumor volume meter (PeiraTM900, Belgium). When the tumor size reached 1200 mm^3^, the tumor tissue was harvested and propagated to the subsequent generation. Once the tumor volumes of PDOX5 and PDOX7 models (*n* = 30 each) reached 50–100 mm^3^, pharmacokinetic studies were carried out. Cantharidin was administered orally to each mouse at a dose of 1 mg/kg. Tumor tissues were collected at 0.5, 1, 2, 4, 8, and 16 h after administration, and intratumoral concentrations of cantharidin were quantified using HPLC analysis.

#### Drug screening *in vivo*

Once the PDOX5 and PDOX7 models (*n* = 15 each) tumor size reached between 50 and 80 mm^3^, mice were randomly divided into the control group (equal amount of normal saline, orally administered; equal amount of DMSO, intraperitoneal injection), cantharidin group (cantharidin, 1 mg/kg/day,^51^ orally administered; equal amount of DMSO, intraperitoneal injection), and cantharidin combined with the Ro67-7476 groups (cantharidin, 1 mg/kg/day, orally administered; Ro67-7476, 4 mg/kg/day,^24^ intraperitoneal injection). Following treatment completion, mice were euthanized via cervical dislocation, and freshly excised tumor tissues were harvested for subsequent experimental analyses. All experimental procedures strictly adhered to the guidelines approved by the Animal Ethics Committee of Tianjin Tian Cheng New Drug Evaluation Co., Ltd (Tianjin, China).

### Method details

#### Construction and culture of CCA PDOs

Immediately after excision, tissue samples were aseptically collected and stored in Advanced DMEM/F12 medium (Gibco, USA) at 4°C for storage and transportation. A tissue fragment approximately 5 mm in diameter was minced and transferred to a 15 mL centrifuge tube containing a digestion solution of Advanced DMEM/F12 medium (Gibco, USA) with 4 mg/mL Collagenase Type IV (Solarbio, China). The sample was digested on a shaker at 37°C for approximately 30–90 min. The residual tissue specimens were divided into two aliquots: one aliquot was cryopreserved at −80°C for subsequent whole-exome sequencing (WES) and transcriptomic profiling, whereas the counterpart aliquot was immediately fixed in 4% paraformaldehyde (PFA) solution for subsequent histopathological examination. The digested tissue was centrifuged at 300×g for 5 min to remove the supernatant, and Matrigel (Corning, USA) was gently mixed with the tumor cell pellet on ice at a 3:1 ratio (Matrigel: PDO medium). The suspension was seeded into 48-well plates at a density of 20,000 cells per 35 μL and incubated at 37°C for approximately 15–30 min to allow Matrigel solidification. After solidification, 300 μL of PDO culture medium was added to each well. The composition of the culture medium for PDO is shown in Table S6. The culture medium was replenished at 2–4 days intervals. When PDOs reached approximately 70%–80% density, they were either passaged at a ratio of 1:2-1:3 or cryopreserved in liquid nitrogen for long-term storage.

#### H&E and histopathology

CCA PDOs were fixed in 4% PFA for 2 h, followed by resuspension in 3% agarose and subsequent solidification on ice. Tumor tissues were also fixed in 4% paraformaldehyde for 24 h. After fixation, both the CCA PDOs and tumor tissues were dehydrated, embedded in paraffin, and sectioned into 3 μm-thick slices. H&E staining was systematically conducted for histological evaluation after deparaffinization and rehydration steps. For IF analysis, tissue sections underwent sequential pretreatment involving 1-h blocking with 5% bovine serum albumin (BSA) and subsequent 10-min membrane permeabilization with 0.1% Triton X-100 solution. The tissue sections were incubated overnight (4°C) with specific primary antibodies, followed by PBS washes. Subsequently, fluorescent-conjugated secondary antibodies were applied for 2 h at ambient temperature. Following additional washing steps, nuclear counterstaining was performed using DAPI-containing mounting medium for nuclei visualization. For IHC evaluation, tissue sections were initially treated with 3% hydrogen peroxide for 15 min to quench endogenous peroxidase activity. Subsequently, non-specific binding sites were blocked by 1-h incubation with 10% goat serum. Primary antibody incubation was performed overnight at 4°C. Following PBS washes, sections were exposed to streptavidin-peroxidase conjugate for 10 min at ambient temperature. After PBS rinsing, species-matched secondary antibodies were applied for 120 min at room temperature, and the sections were sealed using a mounting medium. All images were acquired using a total internal reflection fluorescence microscope (Leica, Germany). Details of the antibodies used in this study are provided in Table S7.

#### WES

The genomic DNA of organoids was isolated, and qualified samples meeting quality control standards were transferred to Novogene Co., Ltd. (Beijing, China) for subsequent library construction and high-throughput sequencing analysis. The raw data were processed and underwent quality control using Fastp (v0.23.1). The cleaned reads were then aligned to the UCSC hg38 reference genome using Bowtie2 (v2.2.9). PCR duplicates were removed from the aligned BAM files with Picard (v2.25.0) by utilizing the “AddOrReplaceReadGroups” and “MarkDuplicates” tools. Local realignment of the reads was done using GATK (v3.8.0) with the “RealignerTargetCreator” and “IndelRealigner” tools. Variant calling was performed with VarScan (v2.3.9), and CNVs were detected using the R package cn. mops (v1.48.0). A set of cancer-related variants was identified by applying the following filters^33^: (1) Variant annotation was initially carried out with snpEff (v2024-04-09) to remove irrelevant sites, including intergenic and intronic regions. (2) To filter out polymorphisms and non-damaging variants we exclude variants which or were included in dbSNP (common _ no _ known _ medical _ impact _ 20170905.vcf) and/or with a frequency >0.01 in ExAC database. (3) Synonymous were also removed. (4) Variants present in COSMIC were retained, and mutations predicted by SIFT to be potentially deleterious to the encoded protein (SIFT score <0.05) were selected.

#### Drug screening and cell viability analysis

CCA PDOs were dissociated into a single-cell suspension using TrypLE Express enzyme (Gibco, USA), and the cells were seeded into a 96-well plate at a density of 2,000 cells per 7 μL of Matrigel for 4 days. After that, PDOs were treated with cantharidin (MedChemExpress, China) (0.01,0.1,1,5,25, and 50 μM), gemcitabine (0.001,0.01,0.1,1,10, and 25 μM) (MedChemExpress, China), cisplatin (MedChemExpress, China) (0.01,0.1,1,5,25, and 50 μM), or adriamycin (Solarbio, China) (0.01,0.1,1,5,25, and 50 μM) for 4 days, and 0.1% DMSO was added as a negative control. Each treatment concentration was replicated across three wells. The cell activity of CCA PDOs was detected by CellTiter-Glo 3D Reagent (Promega, USA), according to the kit’s standard instructions. The results were normalized to the control data and expressed as a percentage of cell viability. Dose-response curves for four chemotherapeutic drugs were plotted using GraphPad Prism 9.0, and the corresponding area under the curve (AUC) and the half-maximal inhibitory concentration (IC50) were calculated. Responses of the CCA PDOs to each chemotherapy drug were categorized into three subgroups: sensitive (lowest 33% AUC), resistant (highest 33% AUC), and intermediate response (middle 34% AUC).^18^^,^^20^

#### RNA-sequencing and transcriptomic analysis

Total RNA was extracted from CCA PDOs across different groups (Control, *n* = 10; cantharidin, *n* = 10) and assessed using the Bioanalyzer 2100 system (Agilent, USA). After library preparation, the samples were sequenced using an Illumina Novaseq X Plus platform. The raw fastq data were initially processed using Fastp (v0.23.1), yielding clean reads for downstream analysis. The reference genome index was generated, followed by alignment of high-quality paired-end reads to the reference sequence utilizing STAR software (version 2.7.8a). FeatureCounts (v1.5.0-p3) were then used to count the number of reads mapped to each gene. The FPKM of each gene was calculated based on gene length and read count. Differentially expressed genes (DEGs) were identified between treated and untreated CCA PDOs using edgeR (v3.42.4), with genes showing a *p*-value <0.05 considered significantly differentially expressed. GO and KEGG enrichment analyses were conducted using the clusterProfiler R package. Gene Set Enrichment Analysis (GSEA) was also performed on the Hallmark and GO gene sets using GSEA (v4.1.0). The raw data of RNA-seq datasets in this study have been deposited in the NCBI Sequence Read Archive, accession number: PRJNA1261625.

#### Network pharmacology analysis

Cantharidin targets were screened using the TCMSP (https://www.tcmspe.com/load_intro.php), HERB (http://herb.ac.cn/), CTD (https://ctdbase.org/), and Swiss Target Prediction databases (https://www. swisstargetprediction. ch/), with the organism set to Homo sapiens and a selection criterion of predicted probability greater than 0, the targets obtained from these databases were merged and deduplicated to derive the predicted targets of cantharidin. Potential CCA-associated targets were retrieved by searching the keyword “Cholangiocarcinoma” in the GeneCards (https://www.genecards.org/), CTD, and TTD databases (https://db.idrblab.net/ttd/). In the GeneCards database, targets with a Relevance Score ≥1 were selected as potential targets for CCA. The intersection between the predicted targets of cantharidin and CCA was analyzed, yielding 685 common targets. These 685 shared target genes were analyzed using the STRING database (https://cn.string-db.org), with the organism set to Homo sapiens and interaction score threshold >0.4, free nodes were removed. The resulting TSV-formatted interaction data were imported into Cytoscape (v3.8.0) for PPI network construction. Core targets were identified through sequentially screening for genes exceeding median values in six centrality measures (Betweenness, Closeness, Degree, Eigenvector Centrality, Network Centrality, and Local Average Connectivity-based Method). Enrichment analysis of the 685 intersection genes was conducted through GO and KEGG analysis, with the results visualized as bubble plots to represent the functional enrichment and pathway analyses.

#### Phosphorylation-ERK1/2 (*p*-ERK1/2) agonist Ro 67-7476

The PDOs were treated with 200 nM *p*-ERK agonist Ro 67–7476^25^ (MedChemExpress, China) for 6 h before cantharidin treatment to activate the *p*-ERK signaling pathway.

#### Western blotting (WB)

Proteins from PDOs and tumor tissues were extracted using cleavage buffers containing RIPA and PMSF (Solarbio, China). The protein samples were quantified using the BCA Protein Assay Kit (Solarbio, China). Following quantification, the samples were boiled for 5 min to denature the proteins. Subsequently, the proteins were loaded onto a 10% SDS-PAGE for electrophoresis. Following electrophoretic separation, the resolved proteins were electro-transferred onto PVDF membranes. The membranes were initially treated with 5% skim milk for blocking at ambient temperature for 1 h, then probed with primary antibodies overnight at 4°C. After TBS-T washes, the membranes were exposed to secondary antibodies for 2 h at room temperature. The specific information on the primary and secondary antibodies is provided in Table S7. The Super ECL substrate (Elabscience, China) was then added to each band, and the exposure intensity was detected using the ChemiDoc MP imaging system (Bio-Rad, USA). Image quantitative testing was performed using ImageJ 7.0.

#### Fluorescein TUNEL staining

The apoptosis levels of PDOs and tumor tissues were assessed using the TUNEL *In Situ* Apoptosis Kit (Elabscience, China). After dewaxing, the slides were incubated with Proteinase K at 37°C for 15 min, followed by treatment with TUNEL-FITC in the dark for 60 min. Subsequently, DAPI staining was performed to counterstain the cell nucleus for 5 min in the dark. The images were captured and analyzed by a total internal reflection fluorescence microscope (Leica, Germany).

#### Flow cytometry

The apoptosis level of each group was determined by Annexin V-FITC/PI Apoptosis Detection Kit (MedChemExpress, China). PDOs were digested with EDTA-free trypsin, then resuspended in 195 μL of binding buffer, followed by sequential addition of 5 μL Annexin V-FITC and 10 μL PI Stain solution. The mixtures were incubated for 15 min at room temperature under light-protected conditions before flow cytometry analysis.

### Quantification and statistical analysis

#### Statistical analysis

Two unpaired continuous variables following the normal distribution were analyzed using the Student’s t test, while comparisons among multiple groups were conducted using a one-way analysis of variance (ANOVA). All statistical analyses were performed using GraphPad Prism software (GraphPad 9.0), with *p* < 0.05 signifying statistical significance. The numerical data are presented as mean ± standard deviation (SD) at least three samples.
