# Supplementary material for: Uncovering cantharidin’s mechanism for cholangiocarcinoma treatment using patient-derived tumor organoids
Source: iScience. 2025 Nov 19;28(12):114136. doi: 10.1016/j.isci.2025.114136 (PMC12719768; doi:10.1016/j.isci.2025.114136)
Supplement: Document S1. Figures S1 and S2 and Tables S1–S7 [file mmc1.pdf]

iScience, Volume *28*

Supplemental information

Uncovering cantharidin's mechanism for cholangiocarcinoma treatment using patient-derived tumor organoids

Pinsheng Han, Libo Wang, Liuyang Zhu, Wen Tong, Sen Liu, Tianze Wang, Tianrun Yang, ZhenZhen Li, Xiaolei Zhou, Tianyu Zhao, Tao Cui, Long Yang, Ze Wang, and Yamin Zhang

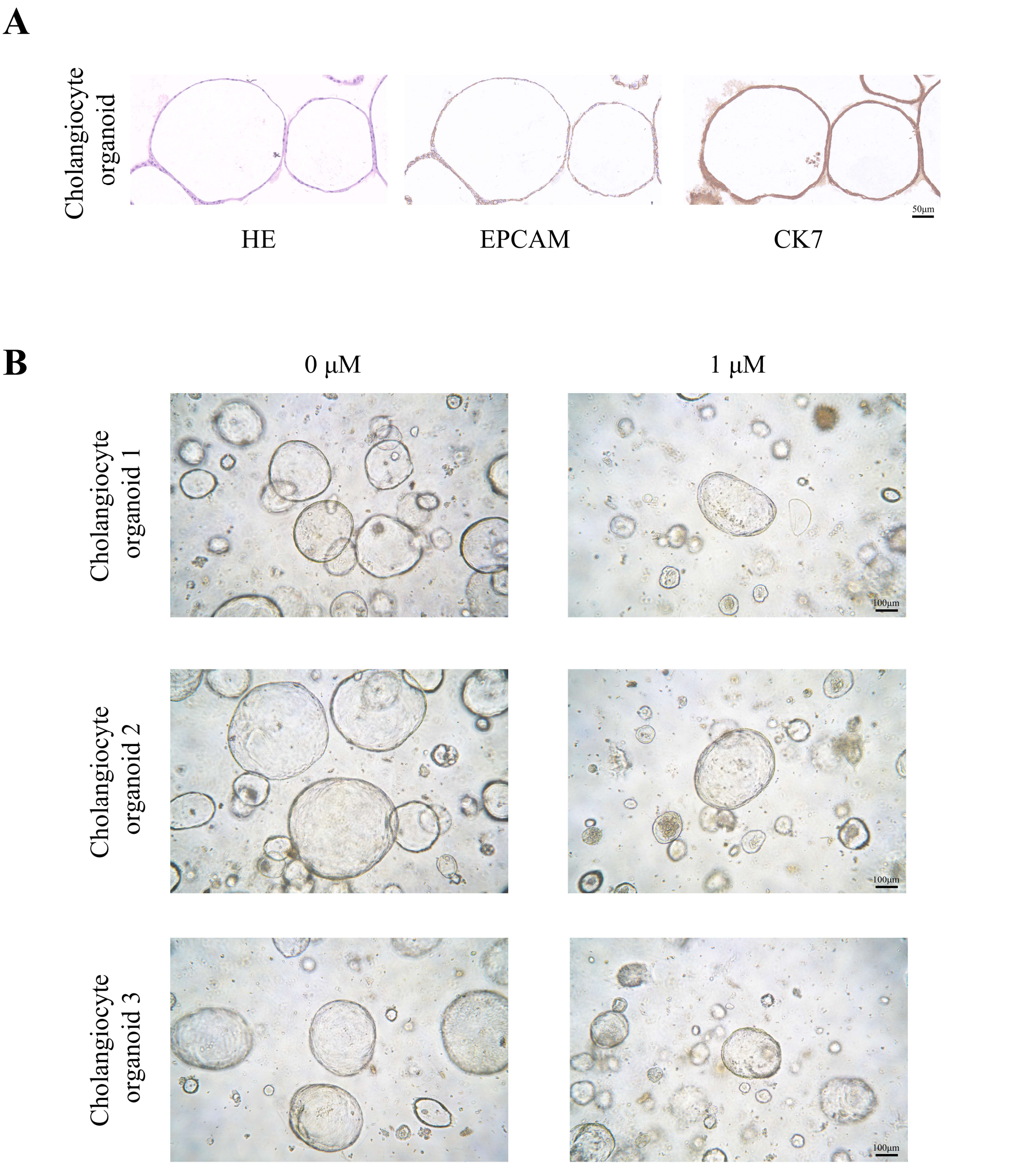

Figure S1. Establishment of normal cholangiocyte organoids and treatment with 1 μM cantharidin (related to Figure 3)

(A) Representative HE and IHC staining images of normal cholangiocyte organoids with EPCAM and CK7. Scale bar, 50 μm.

(B) Representative bright field images of normal cholangiocyte organoids after being treated with 1 μM cantharidin. Scale bar, 100 μm.

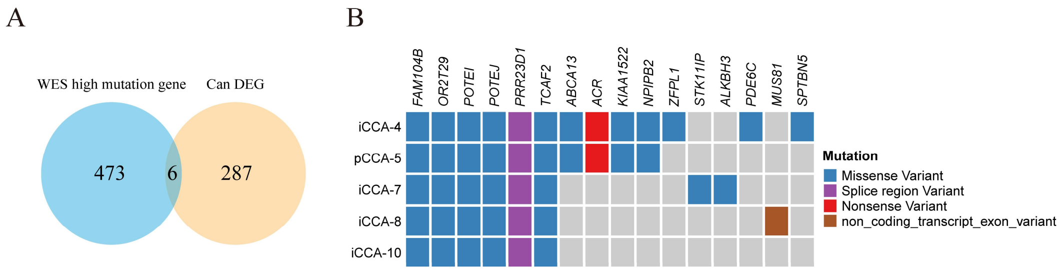

Figure S2. Association between mutated genes in WES, DEGs in cantharidin-treated PDOs and cantharidin sensitivity (related to Figure 2)

(A) Venn diagram of mutated genes in WES and DEGs in Cantharidin-Treated PDOs.

(B) Distribution map of five PDOs shared mutations, mutations in cantharidin-sensitive iCCA-4 and pCCA-5, and shared mutations in WES and DEGs in cantharidin-treated PDOs.

Table S1. The clinical data of CCA patients and CCA pathological characteristics (related to STAR Methods)

Patient ID	Age (y)	Sex	Anatomical subtype	Organoid ID	Histological type	Tumor Stage
1	79	Female	intrahepatic CCA	iCCA-1	Moderately differentiated	T3N1M0 IIIB
2	65	Female	intrahepatic CCA	iCCA-2	Poorly differentiated	T2N0M0 II
3	41	Male	distal CCA	dCCA-3	Moderately differentiated	T2N2M0 IIIA
4	58	Female	intrahepatic CCA	iCCA-4	Moderately differentiated	T1aN0M0 IA
5	49	Female	perihilar CCA	pCCA-5	Poorly differentiated	T2bN1M0 IIIC
6	68	Female	perihilar CCA	pCCA-6	Moderately differentiated	T2bN0M0 II
7	69	Female	intrahepatic CCA	iCCA-7	Moderately differentiated	T2N0M0 II
8	71	Male	intrahepatic CCA	iCCA-8	Poorly differentiated	T3N1M0 IIIB
9	65	Female	distal CCA	dCCA-9	Poorly differentiated	T3N2M0 IIIA
10	72	Male	intrahepatic CCA	iCCA-10	Poorly differentiated	T3N0M0 IIIA
11	42	Male	intrahepatic CCA	iCCA-11	Poorly differentiated	T2N1M0 IIIB
12	61	Male	intrahepatic CCA	iCCA-12	Poorly differentiated	T2N0M0 II
13	60	Female	intrahepatic CCA	iCCA-13	Moderately differentiated	T2N0M0 II
14	49	Female	distal CCA	dCCA-14	Poorly differentiated	T3N0M0 IIb
15	74	Female	distal CCA	dCCA-15	Moderately differentiated	T3N1M0 IIb
16	70	Male	perihilar CCA	pCCA-16	Poorly differentiated	T2bN1M1 IVB

Table S2.WES results in five PDOs with their corresponding primary tissue (related to Figure 2)

iCCA-4	PT4	pCCA-5	PT-5	iCCA-7	PT-7	iCCA-8	PT-8	iCCA-10	PT-10
H6PD	HSPG2	TP73	TP73	PRAMEF17	PRAMEF17	NBPF3	HNRNPR	CALML6	CALML6
HSPG2	MYOM3	PRAMEF1	PRAMEF2	ASAP3	ASAP3	UBXN11	HMGB4	PRAMEF11	PRAMEF4
MYOM3	SYNC	PRAMEF2	PRAMEF2	MAP7D1	MAP7D1	HMGB4	PPIE	PRAMEF10	EPHA10
SYNC	KIAA1522	PRAMEF2	NBPF3	LEXM	LEXM	SUCO	PPIE	PRAMEF10	RLF
KIAA1522	OXCT2	PRAMEF2	UBXN11	NBPF9	NBPF9	NPL	YBX1	EPHA10	WLS
SLC6A9	LEXM	PRAMEF4	KIAA1522	KIF14	KIF14	GUK1	NBPF10	RLF	SORT1
LEXM	C1orf162	PRAMEF10	OXCT2	DDX59	DDX59	OBSCN	NBPF9	WLS	OVGP1
C1orf162	NBPF9	PRAMEF10	SLFNL1	CHIT1	CHIT1	EGLN1	SUCO	SORT1	LORICRIN
NBPF9	ECM1	UBXN11	KPRP	CHIT1	CHIT1	OR2T5	NPL	OVGP1	FCER1A
ECM1	FCER1A	KIAA1522	LORICRIN	NEK2	NEK2	OR2T29	GUK1	NBPF9	F5
FCER1A	SUCO	SLFNL1	OR2T3	PACC1	PACC1	BIRC6	OBSCN	LCE1D	SWT1
SUCO	ASTN1	NBPF9	OR2T5	MTARC1	MTARC1	MTA3	EGLN1	LORICRIN	PDC
ASTN1	SWT1	KPRP	OR2T29	TRIM58	TRIM58	REG3A	OR2T5	MSTO1	DENND1B
SWT1	CHIT1	LORICRIN	PDIA6	OR2T29	OR2T29	POTEI	OR2T29	FCER1A	CHIT1
CHIT1	CHIT1	OR2T3	ARHGEF33	OR2T27	OR2T27	POTEI	BIRC6	F5	CHIT1
OR2T5	OR2T5	OR2T5	SLC3A1	TMEM247	TMEM247	POTEJ	MTA3	SWT1	MIA3
OR2T29	OR2T29	OR2T29	KDM3A	M1AP	M1AP	POTEJ	REG3A	PDC	OR2T5
OR2T27	OR2T27	PDIA6	POTEI	REG3A	THNSL2	NEB	POTEF	DENND1B	OR2T29
HADHB	HADHB	ARHGEF33	POTEJ	THNSL2	NMS	PDE11A	POTEI	CHIT1	NBAS
PCARE	PCARE	SLC3A1	SCN3A	NMS	POTEF	TTN	POTEJ	CHIT1	SH3RF3
DYSF	POTEF	KDM3A	TTN	POTEF	POTEI	TTN	NEB	MIA3	POTEF
REG3A	POTEI	POTEI	SETD2	POTEI	POTEJ	CLK1	CHRNA1	EGLN1	POTEI
ADRA2B	POTEJ	POTEJ	ZNF717	POTEI	AMER3	BARD1	TTN	OR2T3	POTEI
POTEF	MAP3K19	SCN3A	ZNF717	POTEJ	STK11IP	ECEL1	TTN	OR2T5	POTEJ
POTEI	LY75	TTN	ZNF717	AMER3	ZNF717	MTERF4	TTN	OR2T29	ACMSD
POTEJ	LRP2	ZNF717	RETNLB	STK11IP	ZXDC	CCK	BARD1	NBAS	TTN
MAP3K19	ANKZF1	ZNF717	ARHGAP31	ZNF717	RHO	MST1	ECEL1	SH3RF3	TTN
LY75	ECEL1	ZNF717	NR1I2	ZXDC	COL6A5	ZNF717	MTERF4	POTEI	SLC4A3
COBLL1	DLEC1	ZNF717	TNIP2	RHO	AMOTL2	ZNF717	CCK	POTEJ	CELSR3
LRP2	ALS2CL	ZNF717	EVC2	COL6A5	TNK2	AMOTL2	RHOA	ACMSD	TUSC2
TTN	CELSR3	RETNLB	BST1	AMOTL2	ADGRA3	ERICH6	MST1	TTN	POC1A
ANKZF1	MST1	ARHGAP31	ADGRA3	TNK2	ARAP2	PIGX	ZNF717	TTN	ZNF717
ECEL1	ADAMTS9	NR1I2	ARAP2	ADGRA3	HERC5	ARAP2	ZNF717	SLC4A3	CFAP99
ALS2CL	HCLS1	TNIP2	LRRC14B	ARAP2	IQGAP2	ARAP2	BBX	CELSR3	ATP8A1
CELSR3	COL6A6	EVC2	SDHA	HERC5	IQGAP2	TLR1	AMOTL2	TUSC2	SEMA5A
MST1	AMOTL2	BST1	TRIO	IQGAP2	ERAP1	CCSER1	PIGX	POC1A	DNAH5
HCLS1	TNK2	ADGRA3	OTULINL	IQGAP2	IGIP	OTULINL	ARAP2	ZNF717	RICTOR
COL6A6	PIGX	ARAP2	NNT	ERAP1	ARHGAP26	TTC23L	TLR1	CFAP99	CMYA5
AMOTL2	AFM	LRRC14B	GFM2	HMGXB3	HMGXB3	PCDHA4	CCSER1	SEMA5A	HK3
CEP63	SHROOM3	SDHA	FBN2	MGAT1	MGAT1	HLA-A	TTC23L	DNAH5	ATXN1
TNK2	HERC5	TRIO	HLA-B	HLA-B	HLA-B	BBS9	FAM53C	RICTOR	GPX5

PIGX	GALNT7	OTULINL	ADGRG6	CENPW	CENPW	GTF2I	IGIP	CMYA5	OR2H1
USP17L18	PDLIM4	NNT	ABCA13	SUN1	SUN1	PCLO	PCDHA4	HK3	PRRC2A
RPL9	PCDHA11	GFM2	PCLO	PSPH	PCLO	TECPR1	ARHGAP26	ATXN1	HLA-DRB5
AFM	PCDHB5	FBN2	TECPR1	PCLO	TAF6	AHCYL2	ARHGAP26	GPX5	RSPH9
SHROOM3	PCDHB8	HLA-B	CCDC136	TAF6	PAX4	SVOPL	MAPK9	OR2H1	SUN1
HERC5	PCDHB13	ADGRG6	PLXNA4	PAX4	OPN1SW	TCAF2	H2BC12	PRRC2A	GRID2IP
GALNT7	HMGXB3	ABCA13	KIAA1549	OPN1SW	TBXAS1	PRR23D1	FKBP9	RSPH9	PCLO
PRIMPOL	SYCP2L	PCLO	CHPF2	TBXAS1	TCAF2	RP1L1	BBS9	SUN1	TCAF2
SLC6A19	ZKSCAN4	TECPR1	PRR23D1	TCAF2	PRR23D1	DNAAF11	CPSF4	GRID2IP	ZNF862
ADAMTS16	HLA-A	CCDC136	ADGRB1	PRR23D1	C8orf58	DENND3	ATXN7L1	PCLO	PRR23D1
WDR36	HLA-B	PLXNA4	FOXD4L5	C8orf58	PTK2B	FAM205A	WNT2	TCAF2	PRR23D1
PDLIM4	CYP21A2	KIAA1549	CEP78	PTK2B	PREX2	MAPKAP1	AHCYL2	ZNF862	PDLIM2
PCDHA11	HSD17B8	TCAF2	OLFML2A	PREX2	ORM2	C9orf50	SVOPL	PRR23D1	FER1L6
PCDHB5	GPER1	CHPF2	USP20	ORM2	C9orf50	NDOR1	PRR23D1	PRR23D1	DNAAF11
PCDHB8	RPA3	PRR23D1	AGAP4	C9orf50	CEL	PARD3	RP1L1	PDLIM2	KIAA2026
PCDHB13	ABCA13	PRR23D1	AGAP4	CEL	CALML3	OR51A4	DNAAF11	FER1L6	ARID3C
HMGXB3	GALNT17	ADGRB1	IDE	CALML3	PDSS1	TRIM22	DENND3	DNAAF11	FAM205A
SPARC	CALCR	CEP78	CUZD1	PDSS1	OR52N4	LRP4	RC3H2	KIAA2026	ENG
SYCP2L	CBLL1	USP20	MUC5B	OR52N4	IGSF22	PTPMT1	MAPKAP1	GBGT1	ZDHHC12
ZKSCAN4	TMEM168	AGAP4	MUC5B	IGSF22	ALKBH3	TNKS1BP1	C9orf50	BMS1	GBGT1
HLA-A	TCAF2	AGAP4	MOB2	ALKBH3	OR4C46	OR6Q1	NDOR1	WASHC2A	MYO3A
HLA-B	ASB10	IDE	OR51B4	OR4C46	OR8K1	MUS81	PARD3	MUC5B	BMS1
CYP21A2	PRKAG2	CUZD1	OR51B6	OR8K1	OR5M11	VSIG2	MUC5B	OR4C46	WASHC2A
HSD17B8	PRR23D1	MUC5B	OR4C46	OR5M11	ATG16L2	KRT6B	OR51A4	OR5M11	TRIM8
MAP3K7	PDLIM2	MOB2	AHNAK	ATG16L2	CEP295	OR6C70	TRIM22	ZDHHC5	MUC5B
GPER1	RB1CC1	OR51B4	MMP7	CEP295	SLC6A13	DHX37	LRP4	TPCN2	MUC5B
RPA3	FOXD4L5	OR51B6	GPR84	SLC6A13	PRH2	MCF2L	PTPMT1	DHCR7	MUC5B
ABCA13	ROR2	OR4C46	CCER1	PRH2	SLC15A5	PCK2	TNKS1BP1	TRPC6	MUC5B
GALNT17	AOPEP	AHNAK	TMEM119	TAS2R43	KRAS	OR4M2	OR6Q1	PHLDB1	MUC5B
CALCR	ABCA1	MMP7	MTMR6	SLC15A5	CKAP4	OR4M2	MUS81	SLC6A13	MUC5B
CBLL1	PTPN3	GPR84	INF2	KRAS	HIP1R	PHGR1	VSIG2	MANSC1	MUC5B
TMEM168	TTC16	CCER1	CEP170B	CKAP4	DNAH10	PLA2G4B	KRT6B	KRT6B	MUC5B
PTPRZ1	PITRM1	TMEM119	PHGR1	HIP1R	OR11G2	TMC3	HOXC11	GPR84	MUC5B
TCAF2	VIM	INF2	KNL1	DNAH10	MYH6	PPL	OR6C70	EEA1	MUC5B
OR2A14	BMS1	PHGR1	TMC3	OR11G2	FAN1	USP31	DHX37	CCDC168	MUC5B
ASB10	VSTM4	KNL1	RHOT2	MYH6	TRPM1	SMPD3	AKAP11	PAPLN	HPX
PRKAG2	COL13A1	TMC3	SRRM2	KIF26A	GOLGA8B	KRTAP9-7	MCF2L	CIPC	OR4C46
PRR23D1	NPFFR1	NPIPB2	C16orf96	FAN1	PLA2G4D	KRT15	PCK2	AHNAK2	OR5M11
PDLIM2	PDE6C	NPIPA3	NPIPB2	TRPM1	PLA2G4D	KRT9	PHGR1	PLA2G4D	ZDHHC5
ANK1	ARMH3	EARS2	NPIPA3	GOLGA8B	GOLGA6C	MAP3K14	PLA2G4B	PLA2G4D	TPCN2
FOXD4L5	LRP4	KATNIP	EARS2	PLA2G4D	MSLN	DNAH17	TMC3	CILP	DHCR7
ROR2	OR4C46	CES5A	KATNIP	PLA2G4D	MSLN	DNAH17	PPL	MYO9A	TRPC6
ABCA1	OR6Q1	ADGRG3	CES5A	PDCD7	TSR3	MUC16	NPIPA3	TMC3	PHLDB1

PTPN3	OR5B2	MYH10	ADGRG3	GOLGA6C	SRRM2	OR7E24	USP31	RNF151	SLC6A13
TTC16	GANAB	KRTAP1-3	MYH10	MSLN	SLX4	CYP2A7	SMPD3	VWA3A	MANSC1
PITRM1	ZFPL1	KRTAP1-1	KRTAP1-3	MSLN	NPIPA3	ARHGEF1	KRT15	CYB5B	KRT6B
VIM	YIF1A	KRTAP1-1	KRTAP1-1	TSR3	SRCAP	RSPH6A	KRT9	TP53	GPR84
BMS1	TCIRG1	KRTAP4-9	KRTAP1-1	SRRM2	CES5A	PTGIR	MAP3K14	NBR1	EEA1
VSTM4	DHCR7	KRTAP4-4	KRTAP4-9	SLX4	ELMO3	KIR2DL1	TOB1	SDK2	EPSTI1
COL13A1	DYNC2H1	KRT34	KRTAP4-4	SRCAP	SMPD3	KIR2DL1	DNAH17	TUBB8B	CCDC168
NPFFR1	ALG9	ABCA8	KRTAP9-7	CES5A	CLEC18C	TP53TG5	DNAH17	LAMA1	PAPLN
PDE6C	OR6T1	ABCA10	KRT34	ELMO3	RAI1	SS18L1	MUC16	RAB12	CIPC
ARMH3	C1S	OTOP3	ABCA8	SMPD3	KRTAP1-3	LAMA5	OR7E24	PET100	PLA2G4D
LRP4	ACSM4	TMC6	ABCA10	CLEC18C	KRTAP1-1	CBR3	CYP2A7	PRAM1	PLA2G4D
OR4C46	CLEC4E	TJP3	OTOP3	RAI1	KRTAP1-1	KRTAP10-2	ARHGEF1	CCDC105	CILP
OR6Q1	TAS2R43	STAP2	TMC6	KRTAP1-3	KRTAP4-9	RIMBP3B	RSPH6A	CYP2F1	MYO9A
OR5B2	KRT84	ARHGEF1	TJP3	KRTAP1-1	SDK2	LGALS2	PTGIR	NAPSA	TMC3
ZP1	KRT6B	MYH14	STAP2	KRTAP1-1	DNAH17	NAGA	KIR2DL1	KIR2DL1	RNF151
GANAB	OR6C74	SIGLEC12	ARHGEF1	KRTAP4-9	CCDC178	VCX3B	KIR2DL1	KIR2DL1	VWA3A
ZFPL1	NACA	ZNF83	MYH14	SDK2	C18orf63	FAM104B	TP53TG5	KIR3DL1	CYB5B
YIF1A	GPR182	ZNF83	SIGLEC12	DNAH17	PEAK3	SLC25A5	SS18L1	DEFB129	NBR1
TCIRG1	NT5DC3	ZNF83	ZNF83	CCDC178	OR7D2	SLC25A5	LAMA5	BPIFB1	SDK2
DHCR7	MAPKAPK5	KIR2DL1	ZNF83	C18orf63	SUGP2	SLC25A5	CBR3	NRIP1	LAMA1
DYNC2H1	NOS1	KIR3DL1	ZNF83	PEAK3	CYP2F1	SLC25A5	RIMBP3B	KRTAP20-2	RAB12
DYNC2H1	ATP11A	PRNP	KIR2DL1	OR7D2	PLA2G4C	MAGEC1	LGALS2	KRTAP10-2	PET100
ALG9	CUL4A	NRIP1	KIR3DL1	SUGP2	ZNF528	CHRNA7	NAGA	RIMBP3B	PRAM1
PCSK7	CIPC	MAP3K7CL	PRNP	ZNF98	ZNF628		VCX3B	NCF4	CCDC105
OR6T1	CEP128	RIMBP3B	NRIP1	PLA2G4C	DEFB125		FAM104B	SLC16A8	CYP2F1
C1S	AHNAK2	CASTOR1	MAP3K7CL	ZNF528	BPIFA3		CHRNA7	GAGE13	NAPSA
ACSM4	SPTBN5	ACR	RIMBP3B	ZNF628	NRIP1			FAM104B	KIR2DL1
CLEC4E	VPS13C	FAM104B	CASTOR1	DEFB125	MAP3K7CL			PLXNB3	KIR2DL1
TAS2R43	SYNM	PPID	ACR	BPIFA3	DOP1B			NT5C1A	KIR3DL1
KRT84	NPIPB2	ZDHHC11B	FAM104B	NRIP1	DIP2A			NUTM2F	DEFB129
KRT6B	NPIPA3	ZNF107	PPID	MAP3K7CL	AIFM3			NUTM2F	BPIFB1
OR6C74	DNAH3	GOT1L1	INTS1	DOP1B	RGL4				NRIP1
NACA	VWA3A	NUTM2F	ZNF107	DIP2A	ATXN3L				KRTAP20-2
GPR182	ZNF689	NUTM2F	GOT1L1	OR11H1	FAM104B				RIMBP3B
NT5DC3	NDRG4	KRT4	NUTM2F	AIFM3	IDH3G				NCF4
MAPKAPK5	ZDHHC1	CHRNA7	NUTM2F	RGL4	ZNF107				SLC16A8
NOS1	PDF	SERINC4	CHRNA7	ATXN3L	NUTM2F				ACR
ATP11A	SPNS3		SERINC4	FAM104B	NUTM2F				GAGE13
CUL4A	CXCL16			IDH3G					FAM104B
MYH6	ZMYND15			ZDHHC11B					PLXNB3
CIPC	USP6			ZNF107					NT5C1A
CEP128	GPS2			NUTM2F					POLN
AHNAK2	CCDC144A			NUTM2F					NUTM2F

SPTBN5	ETV4			SELPLG					NUTM2F
PLA2G4F	LOXHD1			CHRNA7					NCOR2
ATP8B4	ATP9B								
VPS13C	OR7D2								
SYNM	ADGRE3								
NPRL3	WIZ								
NPIPB2	CHST8								
DNAH3	CYP2A7								
VWA3A	CYP2F1								
ZNF689	CYP2F1								
NDRG4	TULP2								
ZDHHC1	HAS1								
SPNS3	MAP3K7CL								
CXCL16	RRP1								
ZMYND15	KRTAP10-2								
USP6	OR11H1								
GPS2	CLTCL1								
MYH8	RIMBP3B								
CCDC144A	MYO18B								
KRTAP17-1	TRIOBP								
ETV4	ACR								
TANC2	VCX3B								
DNAH17	DDX53								
LOXHD1	ZNF182								
ZNF516	FAM104B								
ATP9B	PCDH11X								
OR7D2	PCDHGA6								
ADGRE3	KRT4								
WIZ									
RASAL3									
CHST8									
CYP2A7									
CYP2F1									
CYP2F1									
TULP2									
HAS1									
LILRA4									
MAP3K7CL									
RRP1									
KRTAP10-2									
OR11H1									
CLTCL1									
RIMBP3B									

MYO18B									
TRIOBP									
ACR									
DDX53									
ZNF182									
FAM104B									
KLHL4									
PCDH11X									
SLC4A3									
PCDHGA6									

Note that some genes are listed multiple times due to mutations occurring at different loci.

Table S3:The diameter(μm) of CCA PDOs after administration (related to Figure 3)

Diameter（μm）	Cantharidin							Gemcitabine						
Drugs Concentration	0μM	0.01μM	0.1μM	1μM	5μM	25μM	50μM	0μM	0.001μM	0.01μM	0.1μM	1μM	10μM	25μM
iCCA-1	198	163	117	93	34	33	30	198	118	78	68	57	33	32
iCCA-2	150	154	151	114	33	35	28	150	125	58	45	37	35	29
dCCA-3	148	142	117	83	32	27	27	148	115	95	53	39	32	27
iCCA-4	105	49	38	36	33	31	29	105	45	41	37	36	31	28
pCCA-5	82	49	40	39	35	33	29	120	124	56	43	34	32	33
pCCA-6	218	187	157	93	37	32	30	218	213	201	137	71	68	33
iCCA-7	120	124	108	48	39	38	27	82	59	38	36	36	30	29
iCCA-8	238	227	201	138	57	33	30	238	237	167	118	73	48	37
dCCA-9	187	179	146	133	62	33	31	187	137	77	39	33	30	26
iCCA-10	108	102	84	61	32	28	25	108	99	88	53	37	34	32
iCCA-11	117	108	97	91	57	43	37	117	107	96	73	67	54	43
iCCA-12	143	139	117	100	74	35	32	143	144	138	129	107	96	37
iCCA-13	274	269	264	67	58	48	31	274	248	196	168	127	85	57
dCCA-14	287	252	196	102	73	56	29	287	282	178	156	102	75	46
dCCA-15	206	195	185	173	52	37	36	206	207	174	126	82	64	33
pCCA-16	186	161	130	83	38	32	31	186	132	82	70	64	45	36
Diameter（μm）	Cisplatin							Adriamycin						
Drugs Concentratio	0μM	0.01μM	0.1μM	1μM	5μM	25μM	50μM	0μM	0.01μM	0.1μM	1μM	5μM	25μM	50μM
iCCA-1	198	195	167	117	98	76	64	198	145	115	86	79	39	34
iCCA-2	150	148	137	78	48	43	29	150	97	38	34	31	27	29
dCCA-3	148	99	67	59	49	43	37	148	78	48	32	31	29	25
iCCA-4	105	99	85	73	62	40	37	105	39	35	27	25	27	24
pCCA-5	120	117	108	93	47	37	26	120	113	97	38	36	34	27
pCCA-6	218	207	202	167	128	84	54	218	175	147	82	38	32	29
iCCA-7	82	77	69	67	46	46	39	82	68	59	35	27	25	24
iCCA-8	238	239	235	175	117	67	36	238	127	99	67	33	30	27
dCCA-9	187	159	133	78	65	37	30	187	124	74	37	33	32	30
iCCA-10	108	109	104	99	91	62	31	108	107	88	62	37	32	29
iCCA-11	117	116	102	103	74	64	53	117	98	85	67	48	37	32
iCCA-12	143	137	124	107	97	93	34	143	114	91	57	48	35	33
iCCA-13	274	262	248	234	197	164	57	274	257	249	181	62	37	32
dCCA-14	287	265	248	197	157	149	51	287	237	185	92	54	38	32
dCCA-15	206	201	204	193	188	131	47	206	195	187	166	142	57	43
pCCA-16	186	181	172	149	131	103	62	186	141	107	77	36	32	30

Table S4. The IC50 of CCA PDOs for the different treatment groups (related to Figure 3)

Organoid ID	Cantharidin	Gemcitabine	Cisplatin	Adriamycin
iCCA-1	0.14530	0.00046	1.21000	0.11820
iCCA-2	1.26200	0.00350	0.93220	0.01426
dCCA-3	0.57490	0.01693	0.03548	0.01186
iCCA-4	0.00706	0.00001	3.82200	0.00317
pCCA-5	0.01160	0.00830	3.29300	0.22100
pCCA-6	0.26610	0.15980	5.58100	0.16710
iCCA-7	0.80050	0.00224	1.63100	0.18420
iCCA-8	0.96060	0.06556	2.82700	0.01731
dCCA-9	0.84650	0.00201	0.24700	0.01426
iCCA-10	0.75890	0.13500	16.39000	0.87620
iCCA-11	1.65000	0.48160	8.31800	0.49070
iCCA-12	1.51300	6.25800	7.50900	0.17780
iCCA-13	0.59550	0.14240	10.47000	1.02700
dCCA-14	0.15300	0.04997	2.65600	0.08661
dCCA-15	1.57800	0.18350	23.11000	4.18600
pCCA-16	0.18450	0.00214	7.07200	0.08069
Average	0.70672	0.46946	5.94398	0.47977

Table S5. The clinical outcomes of CCA patients who received adjuvant chemotherapy following surgery (related to Figure 3)

Organiod ID	Date of operation	Postoperative chemotherapy regimens	Clinical response	Clinical respons	Organoid treatment	Correlation
iCCA-2	20220419	GP (Gemcitabine+Cisplatin) *6	No recurrence（20231205）	Sensitive	Sensitive<br>Intermediate sensitive	yes
dCCA-9	20220608	GP (Gemcitabine+Cisplatin) *6	No recurrence（20240313）	Sensitive	Sensitive<br>Intermediate sensitive	yes
iCCA-11	20220615	GP (Gemcitabine+Cisplatin) *4	Recurrence（20230517）	Resistant	Intermediate sensitive<br>Resistant	yes
iCCA-12	20220708	GP (Gemcitabine+Cisplatin) *4	Recurrence（20230315）	Resistant	Resistant<br>Resistant	yes
dCCA-14	20220919	GP (Gemcitabine+Cisplatin) *4	No recurrence（20240614）	Sensitive	Intermediate sensitive<br>Intermediate sensitive	yes

Table S6. Composition of CCA organoid medium (related to STAR Methods)

The culture medium for PDO	Company	Cat No.	Concentration
Advanced DMEM/F-12	Gibco	12634010	-
Penicillin-streptomycin	Gibco	15140122	1%
GlutaMax	Gibco	35050061	1%
HEPES	Gibco	15630106	10mM
B27 supplement without vitamin A	Gibco	A3353501	1:50
N2 supplement	Gibco	17502048	1:100
N-Acetyl-L-cysteine	MedChemExpress	HY-110256	1.25mM
Nicotinamide	MedChemExpress	HY-B0150	10mM
Gastrin I	MedChemExpress	HY-P1097	10nM
Forskolin	MedChemExpress	HY-15371	10μM
A83-01	MedChemExpress	HY-10432	5μM
Y-27632	MedChemExpress	HY-10071	10mM
Recombinant human EGF	Novoprotein	C029	50ng/ml
Recombinant human FGF10	Novoprotein	CR11	100ng/ml
Recombinant human HGF	Novoprotein	CJ72	25ng/ml
Recombinant Human Noggin	Novoprotein	CB89	25ng/ml
Recombinant Human R-spondin 1	Novoprotein	CX83	200ng/mL
Recombinant Human Wnt3a	Novoprotein	C22R	100ng/mL

Table S7. Details of the antibodies used in this manuscript (related to STAR Methods)

Target	Company	Cat No.	Dilution
EPCAM	Proteintech	66316-1-Ig	IF:1:200; IHC:1:400
CK7	Abcam	ab181598	IF:1:100; IHC:1:5000
Phospho-ERK1/2	Affinity	AF1015	WB:1:1000; IF:1:200
ERK1/2	Proteintech	11257-1-AP	WB:1:3000
c-Fos	Proteintech	66590-1-Ig	WB:1:5000
Bax	Proteintech	50599-2-Ig	WB:1:3000
Bcl-xl	Proteintech	26967-1-AP	WB:1:1000
Cyclin D1	Proteintech	60186-1-Ig	WB:1:5000
GRP78	Proteintech	11587-1-AP	WB:1:2000
CHOP	Proteintech	15204-1-AP	WB:1:1000
GAPDH	Proteintech	60004-1-Ig	WB:1:50000
Ki67	Proteintech	27309-1-AP	IF:1:100
Goat Anti-Mouse Secondary Antibody	Proteintech	RGAM001	WB:1:3000
Goat Anti-Rabbit Secondary Antibody	Proteintech	RGAR001	WB:1:3000
Goat Anti-Rabbit IgG H&L (Alexa Fluor® 594)	Abcam	ab150080	IF:1:200
Goat Anti-Mouse IgG H&L (Alexa Fluor® 488)	Abcam	ab150113	IF:1:200
